# Transcriptional Activation Mechanisms and Target Genes of the Oncogene Product Tax of Human T-Cell Leukemia Virus Type 1

**DOI:** 10.3390/genes16101221

**Published:** 2025-10-15

**Authors:** Mashiro Shirasawa, Rinka Nakajima, Yaxuan Zhou, Mariana Fikriyanti, Ritsuko Iwanaga, Andrew P. Bradford, Kenta Kurayoshi, Keigo Araki, Kiyoshi Ohtani

**Affiliations:** 1 Department of Biomedical Sciences, School of Biological and Environmental Sciences, Kwansei Gakuin University, 1 Gakuen Uegahara, Sanda 669-1330, Hyogo, Japan; icf08267@kwansei.ac.jp (M.S.); hnj51097@kwansei.ac.jp (R.N.); gtk53096@kwansei.ac.jp (Y.Z.); hsj19688@kwansei.ac.jp (M.F.); 2 Department of Obstetrics and Gynecology, University of Colorado School of Medicine, Anschutz Medical Campus, 12700 East 19th Avenue, Aurora, CO 80045, USA; ritsuko.iwanaga@cuanschutz.edu (R.I.); andy.bradford@cuanschutz.edu (A.P.B.); 3 Division of Molecular Genetics, Cancer Research Institute, Kanazawa University, Kakuma-machi, Kanazawa 920-1192, Ishikawa, Japan; kuraken0901@gmail.com; 4 Department of Morphological Biology, Ohu University School of Dentistry, 31-1 Misumido Tomitamachi, Koriyama 963-8611, Fukushima, Japan; k-araki@den.ohu-u.ac.jp

**Keywords:** HTLV-1, Tax, *trans*-activation, target genes, leukemogenesis

## Abstract

Human T-cell leukemia virus type 1 (HTLV-1) is the causative agent of adult T-cell leukemia/lymphoma (ATL). The trans-activator protein Tax of HTLV-1 is thought to play a crucial role in the early-stage transformation of the virus-infected cells. Tax is a multi-functional protein and modulates cellular signaling pathways that promote proliferation and survival of HTLV-1-infected cells, primarily through the trans-activation of cellular target genes. Tax interacts with a variety of host cell factors including signal transducers and transcription factors, leading to the activation of transcription factors such as CREB, NF-κB, and SRF and activates both its own promoter and those of a variety of host cellular genes. Tax activates its own promoter mainly through CREB and host cellular genes through NF-κB, SRF, and CREB. Accumulating evidence indicates that the Tax-mediated trans-activation of target genes through NF-κB plays an essential role in the transformation of HTLV-1 infected cells. However, the repertoire of Tax target genes, especially those crucial for leukemogenesis, are not known in detail. In this review, we summarize transcriptional activation mechanisms and target genes of Tax, especially focusing on transformation, to facilitate understanding of the underlying mechanisms of leukemogenesis induced by HTLV-1 infection.

## 1. Introduction

Human T-cell leukemia virus type 1 (HTLV-1) is the first retrovirus discovered in humans [1,2] and is the causative agent of the unique type of leukemia/lymphoma, adult T-cell leukemia/lymphoma (ATL) [3,4,5,6]. ATL was first reported in Japan [7] and is characterized by adult onset leukemic cells with a mature helper-T phenotype (CD4+, CD25+) and indented nuclei, frequent skin infiltration of leukemic cells, hypercalcemia, and a striking geographic clustering of the patients in the south-western part of Japan [8]. Other ATL endemic areas include sub-Saharan Africa, the Caribbean coast, South America, and parts of the Middle East and Australia [9]. Geographic clustering suggested infectious agents as the cause of ATL. Accordingly, a type C retrovirus particle was identified by electron microscopy in an ATL cell line, and all ATL patients, without exception, possessed antibodies for the virus, establishing that the infection of HTLV-1 is the cause of ATL [3,5]. HTLV-1 has also been isolated from fresh and cultured lymphocytes of a patient with cutaneous T-cell lymphoma, but causality in that disease has not been addressed [1]. The isolated HTLV-1 virus by itself cannot infect target cells efficiently and is mostly transmitted from cell to cell [10,11,12,13,14]. The virus is mainly transmitted from mother to child through the virus-infected cells in breast milk. About 5–10 million people worldwide are estimated to be infected with HTLV-1 and approximately 2–5% of virus-infected people develop ATL after a long latency (40–50 years) [9]. HTLV-1 infection has also been shown to cause HTLV-1-associated myelopathy (HAM)/tropical spastic paraparesis (TSP) and other inflammatory diseases [6,15,16,17,18].

**Figure 1:**
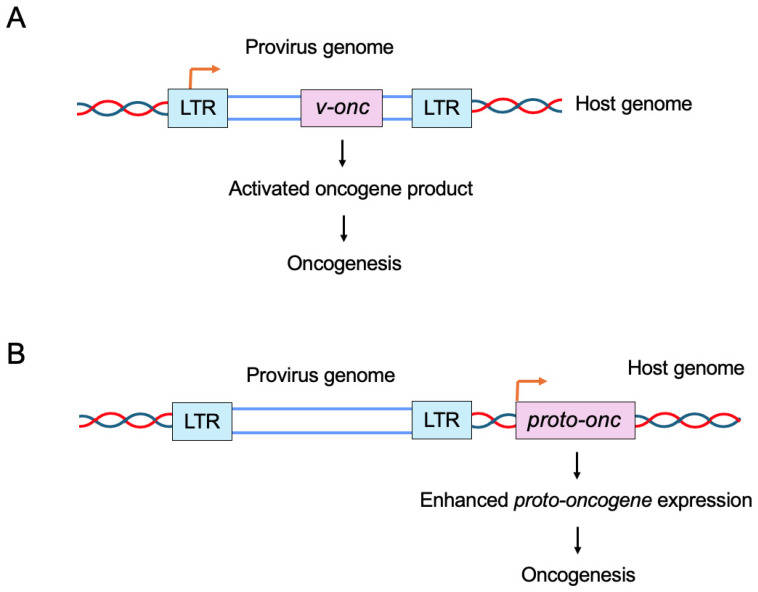
Mechanisms of oncogenesis induced by animal retroviruses. (**A**) Introduction of a viral oncogene (*v-onc*). The virus possesses an activated oncogene derived from a cellular proto-oncogene and introduces the activated oncogene into the host cell genome upon infection. (**B**) Promoter insertion into or near a host proto-oncogene. When the virus genome is inserted into or near a host cell proto-oncogene, it enhances the expression of that gene due to promoter activity of the viral LTR.

Animal retroviruses transform host cells by two mechanisms, as previously reported (Figure 1) [19,20,21]. One is the transduction of viral oncogenes (*v-onc*) derived from cellular proto-oncogenes. The other is the insertion of the viral genome, which contains a promoter to drive its own gene expression, within or in close proximity to proto-oncogenes of host cells, leading to an increased expression of those genes.

**Figure 2:**
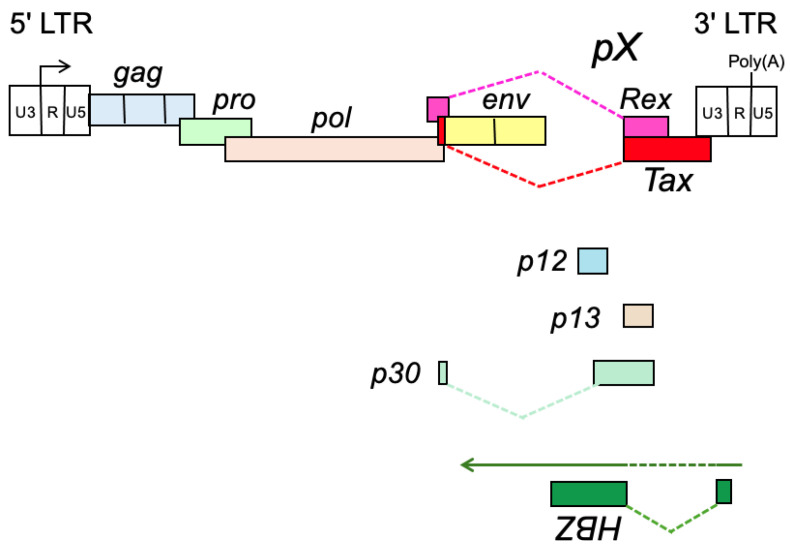
Organization of the HTLV-1 genome. In addition to common structures of retroviral genomes such as LTRs, *gag*, *pro*, *pol* and *env*, the HTLV-1 genome contains a *pX* region, which lacks homology to the mammalian genome and codes for several regulatory proteins, such as Tax and Rex. An additional regulatory protein HBZ is encoded in the anti-sense strand.

The HTLV-1 genome exhibits features and an organization in common with other retroviruses, such as long terminal repeats (LTRs), *gag*, *pro*, *pol*, and *env* genes, but, unlike oncogenic animal retroviruses, it does not contain viral oncogenes derived from cellular proto-oncogenes [22]. In addition, the virus integration sites are not accumulated in the vicinity of certain host proto-oncogenes [23]. Thus, the two mechanisms of oncogenic animal retroviruses do not apply to HTLV-1-mediated oncogenesis. The HTLV genome has a unique *pX* region (protein coding region X) between *env* and 3′ LTR, which codes for several regulatory proteins such as the trans-activator of *pX* region (Tax), Rex, p30, p13, and p12 (Figure 2) [5,24,25,26].

**Figure 3:**
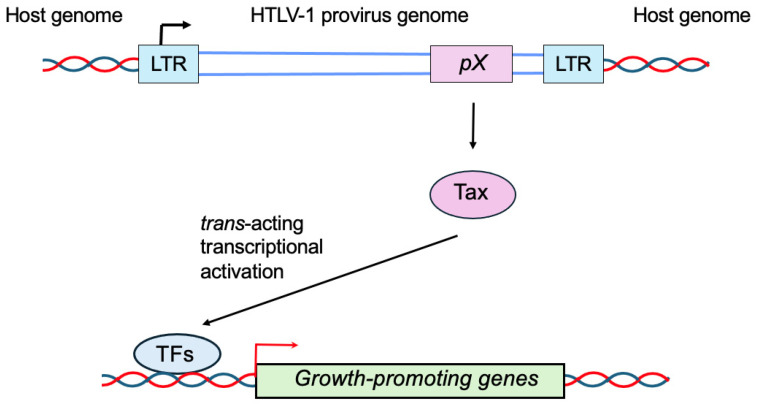
The mechanism of oncogenesis exploited by HTLV-1. The *trans*-acting transcriptional activation mechanism. Tax encoded in the *pX* region functions as a transcriptional activator, which activates cellular transcription factors (TFs) to induce the expression of growth-promoting genes to facilitate the growth of host T cells.

Tax functions as a transcriptional regulator and plays a crucial role in the expression of viral genes, and Rex is important in determining the expression of structural proteins by suppressing the splicing of transcribed viral mRNA. An additional regulatory protein, HTLV-1 basic leucine zipper factor (HBZ), is encoded in the anti-sense strand, which plays a crucial role in the persistence of virus-infected cells. Among these viral regulatory proteins, Tax is thought to play crucial roles in the early-stage transformation of HTLV-1-infected cells by *trans*-activating cellular genes regulating proliferation and survival (Figure 3) [5].

Tax is a multifunctional protein that interacts with a variety of cellular factors and modulates transcription pathways to activate viral and host cell gene expression, promotes cell cycle progression, facilitates cell survival, and induces genome instability [5,6,27,28,29,30,31,32]. Tax facilitates virus replication by functioning as a transcriptional activator for its own promoter in the 5′ LTR through activation of the CREB/ATF family of host cell transcription factors [33,34]. Tax also trans-activates a variety of cellular genes involved in cell proliferation and survival through the activation of cellular transcription factors, such as NF-κB and SRF, and is thought to play a crucial role in the early stage of the transformation of virus-infected cells [5,6,27,28,29,30,31,32]. Among cellular transcription factors activated by Tax, NF-κB is thought to be critical for the transformation of the virus-infected cells [28,35,36,37]. Indeed, the ability of Tax to immortalize peripheral blood lymphocytes (PBLs) is dependent on the NF-κB pathway and the presence of interleukin (IL-2) [38,39,40,41,42,43]. However, Tax protein is highly immunogenic, and its expression is maintained at extremely low levels in ATL cells [44], likely to evade host immune surveillance. In addition, the sustained activation of NF-κB by Tax induces cellular senescence [37,45,46]. To maintain the expression of Tax at low levels and to avoid cellular senescence induced by the sustained activation of NF-κB, the HTLV-1 bZIP factor (HBZ), encoded by the anti-sense strand, counteracts many functions of Tax to permit the persistence of virus-infected cells [27,37,45,47,48,49,50,51,52]. HBZ suppresses Tax-mediated activation of the LTR by interacting with CREB/ATF family transcription factors, reducing the expression of Tax to escape immune surveillance. HBZ also suppresses Tax-mediated activation of the canonical NF-κB pathway by suppressing the DNA-binding capacity of p65 and by facilitating the degradation of p65 [49]. p30 and p13 also suppress HTLV-1 gene expression by sequestering Tax coding mRNA in the nucleus and interfering with the Tax-CBP/p300 interaction [53,54,55,56,57,58].

Although Tax plays a central role in the promotion of and proliferation of virus-infected cells, not all virus carriers progress to ATL. About 2–5% of the virus-infected population develop ATL, and additional genetic changes are thought to be required for malignant transformation. Tax induces genome instability by the trans-activation of target genes and inhibition of the cell cycle and DNA damage checkpoints through interacting with the mediators of those signal transduction pathways. This leads to the accumulation of mutations in the virus-infected cells [59,60,61], which may contribute to malignant transformation [62,63].

Since Tax immortalization of peripheral blood T cells depends on its ability to activate NF-κB [40,41,42,43], the Tax-mediated activation of cellular genes through NF-κB is thought to be crucial for the transformation of host T cells by HTLV-1 infection [28,35,36,37]. However, the repertoire of Tax target genes, especially those crucial for leukemogenesis, are not known in detail [31]. In this review, we will summarize transcriptional activation mechanisms and target genes of Tax to facilitate the understanding of the mechanisms of leukemogenesis induced by HTLV-1 infection. Other integrated reviews have focused on the protein–protein interactions of Tax and their consequent downstream effects [27,28,30,31,32,36,64].

## 2. Roles of Tax in HTLV-1-Mediated Transformation

A variety of experimental data support the notion that Tax plays a central role in the early-stage transformation of virus-infected cells [6,27,28,29,30,31,32,65]. Virus vector-mediated introduction of Tax in normal peripheral blood T cells immortalized the cells dependent on the T-cell growth factor IL-2 [38,39]. Immortalization of peripheral blood T lymphocytes by HTLV-1 Tax mutants, introduced by the retrovirus vector or a functional molecular clone, shows that the ability of Tax to activate the NF-κB pathway is necessary and sufficient for the immortalization of normal T cells, whilst the CREB pathway is dispensable [40,41,42,43]. The introduction of Tax into immortalized mouse and rat fibroblast cell lines causes phenotypic transformation and renders the rat cell line tumorigenic in nude mice [66,67,68]. Tax has the ability to promote cell cycle progression depending on its ability to activate the NF-κB pathway [69,70,71]. Tax-transgenic mice developed a variety of tumors including mesenchymal tumors [72,73,74] and leukemia/lymphoma [75,76,77,78], depending on the promoter used [79,80]. In a murine ATL model, triggering Tax proteolysis cures Tax-driven ATL in mice by reducing the tumor-initiating cells [81]. Although Tax expression is very low in vivo, Tax is expressed in a small fraction of leukemic cells and spontaneously switches between on and off states [82]. The transient expression of Tax confers anti-apoptotic activity by upregulating anti-apoptotic gene expression, and this anti-apoptotic effect continues even if Tax expression diminished. This activation creates an antiapoptotic environment that extends to nearby Tax-negative cells, allowing the whole population of cells to survive [82]. The knockdown of Tax rapidly induced apoptosis in most cells, indicating that the transient expression of Tax is critical for maintaining the whole population of the virus-infected cells [82]. Similarly, primary ATL cells from patients with acute or chronic ATL express very low levels of Tax mRNA and protein, but survival of these primary ATL cells is dependent on continued Tax expression [83]. Accordingly, the downregulation of Tax reduced NF-κB activity and resulted in p53/PML activation and apoptosis [83]. Simian T-lymphotropic Virus (STLV) is the etiological agent of ATL in non-human primates, serving as a crucial animal model for studying HTLV-1 pathogenesis [84]. Tax proteins of HTLV-1 and STLV-1 are highly homologous and fulfill similar functions [85,86], supporting the crucial roles of Tax in pathogenicity, although subtle differences in function exist, such as the HTLV-1 Tax showing higher transforming activity than STLV-1 Tax, partly due to its more efficient activation of the non-canonical NF-κB pathway [84]. In addition, recent pilot clinical studies show the effectiveness of the Tax peptide-pulsed dendritic cell (DC) vaccine for treatment of ATL [87,88,89], suggesting that Tax is indeed expressed in ATL cells in vivo and serves as a target of immunotherapy. These observations suggest that Tax may also play important roles in later stages of leukemogenesis induced by HTLV-1 infection.

## 3. Tax Activates Host Cell Transcription Factors

Tax transcriptionally *trans*-activates not only its own promoter to augment the virus gene expression [33,34,90,91] but also a variety of host cell genes to promote cell proliferation and survival [5,6,27,28,29,30,31,32]. Tax interacts with a variety of cellular factors to activate at least three transcription factors, cAMP responsive element (CRE)-binding factor (CREB), nuclear factor (NF)-κB, and serum-responsive factor (SRF), to regulate target genes [5]. Intriguingly, while CREB is important for the activation of HTLV-1 gene expression, it is the Tax-induced activation of NF-κB that plays a critical role in the immortalization of host T cells [28,31,37].

### 3.1. cAMP Responsive Element-Binding Factor (CREB)/Activating Transcription Factor (ATF)

Tax increases HTLV-1 gene expression by activating the viral promoter in the long terminal repeat (LTR) [33,34,90,91]. Tax *trans*-activates the HTLV-1 LTR primarily via the 21 bp-motifs present in three tandem repeats in the U3 region of LTR [92,93,94]. Tax has a marginal affinity for DNA and does not directly bind to the target sequences [95]. The 21 bp-motifs contain a cyclic AMP response element (CRE), which is activated by cAMP through the CRE binding protein (CREB) [96]. CREB is a member of a family of basic leucine zipper (bZIP) transcription factors in which the leucine zipper is utilized for the formation of homo- or heterodimers, and the basic region mediates binding to the target DNA, cAMP response elements (CRE). CREB forms homo- or heterodimers with other bZIP transcription factors, such as activating transcription factors (ATFs). The activity of CREB is normally regulated by phosphorylation by protein kinase A or Ca^2+^/calmodulin-dependent protein kinases on Ser133 (pCREB), resulting in the recruitment of the transcriptional coactivator CREB-binding protein (CBP)/p300 for transcriptional activation.

**Figure 4:**
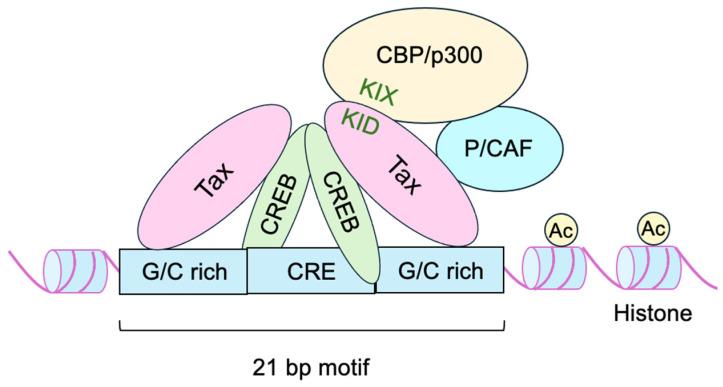
Tax activates HTLV-1 LTR primarily via CREB. Tax indirectly binds to CRE of 21 bp-motifs in HTLV-1 LTR via CREB, while recognizing GC-rich sequences flanking CRE. Tax recruits transcriptional coactivators such as CBP/p300 and P/CAF to activate transcription. KID: kinase-inducible domain (KID)-like domain, KIX: kinase-inducible domain (KID) interacting domain, and Ac: acetylation.

Tax binds indirectly to the 21 bp-motifs through CREB and stabilizes CREB binding to DNA (Figure 4) [95,97]. Tax also binds to ATF2 and CREB-2 (ATF-4) and stabilizes their binding to the 21 bp-motifs to activate transcription [98,99,100]. Tax binds to a basic segment in the bZIP of CREB and ATF, thereby enhancing their affinity for the target sequence [101]. Although Tax enhances CREB binding to the 21 bp-motifs, Tax does not enhance the binding of CREB to other cellular CREs, such as the somatostatin CRE [102,103]. Tax-mediated enhancement of CREB binding to the 21 bp-motifs is dependent on the GC-rich flanking sequences of the viral CRE, with which Tax interacts directly to stabilize CREB binding (Figure 4) [104,105]. Accordingly, Tax does not activate all CRE-containing cellular genes. SR-related protein TAXREB803/SRL300 binds to this GC-rich flanking sequence and contributes to the activation by Tax [106].

Tax bound to CREB recruits CBP/p300, independent of CREB phosphorylation, to activate transcription [95,107,108,109,110]. The interaction between Tax and CBP/p300 is mediated through the kinase-inducible domain (KID)-like domain of Tax [111,112] and multiple regions of CBP/p300, including the KIX, C/H1, and SRC-1-interacting domains (Figure 4) [108,113,114,115]. Tax also interacts with the transducers of regulated CREB 1 (TORC1), TORC2, and TORC3 and enhances p300 recruitment [116,117].

CREB is highly phosphorylated in HTLV-1-infected T-cell lines, and phospho-CREB plays an important role in activation of the LTR by Tax [118]. Tax also binds PCAF and activates the transcription independent of its HAT activity (Figure 4) [119]. Recruitment of CBP/p300 facilitates the acetylation of histones around the viral promoter and loosens chromatin structure in co-operation with the histone chaperone, nucleosome assembly protein 1 (NAP1), to promote transcription (Figure 4) [120,121]. Tax also recruits the chromatin remodeling factor BRG1 to facilitate transcription [122]. In addition, Tax binds to histone deacetylase HDAC1 and inhibits binding to and/or promotes its dissociation from the HTLV-1 promoter [123], thereby attenuating suppression of transcription by deacetylation. Myocyte enhancer factor (MEF)-2 stabilizes Tax/CREB binding to target sequences by binding to the HTLV-1 LTR [124].

### 3.2. Nuclear Factor-Kappa B (NF-κB)

In contrast to the HTLV-1 LTR, which is mainly activated by Tax through CREB, many cellular genes important for the transformation of virus-infected cells are activated by Tax via the transcription factor NF-κB.

NF-κB plays pivotal roles in important cellular processes such as cell survival, proliferation, and immune responses. The NF-κB family is composed of RelA/p65, RelB, c-Rel, p50, and p52, which are structurally related. The NF-κB proteins activate target gene expression by binding to NF-κB response elements in the promoter regions of target genes as heterodimers (RelA/p50, c-Rel/p50, and RelB/p52) [125]. Whereas RelA/p50 preferentially binds to the sequence 5′-GGGRNNYYCC-3′ (R = purine, N = any nucleotide, and Y = pyrimidine), RelB/p52 recognizes the sequence 5′-RGGAGAYTTR-3′ that is not bound by the RelA:p50 dimer [126].

In the absence of activating stimuli, the NF-κB proteins are retained in the cytoplasm by binding to inhibitory molecules, the inhibitor of NF-κB (IκBα, IκBβ, and IκBε) for RelA/p50 and c-Rel/p50, and p100 (precursor of p52) for RelB/p52, and are kept transcriptionally inactive. The activation of NF-κB proteins are mediated through the canonical and non-canonical pathways [125,127].

Classical NF-κB proteins (RelA/p50 and c-Rel/p50 heterodimers) are activated through the canonical pathway. Upon activating stimuli, the IκB kinase (IKK) complex is activated and phosphorylates IκB, which leads to ubiquitination and proteasome-mediated degradation [128]. This releases NF-κB and unmasks a nuclear localization signal, resulting in the translocation of NF-κB into the nucleus and activation of target genes [125,129,130,131]. The IKK complex is composed of two catalytic subunits (IKKα and IKKβ) and a regulatory subunit (NF-κB essential modulator (NEMO) or IKKγ) [128].

In contrast, RelB/p52 is activated through the non-canonical NF-κB pathway. RelB/p100 (p52 precursor) is retained in the cytoplasm through the action of the ankyrin motif of p100. In response to stimuli, NF-κB-inducing kinase (NIK) activates IKKα, which phosphorylates p100. This phosphorylation marks p100 for ubiquitination and limited proteolysis to generate mature p52, leading to the translocation of RelB/p52 into the nucleus and activation of target genes [127,132,133].

**Figure 5:**
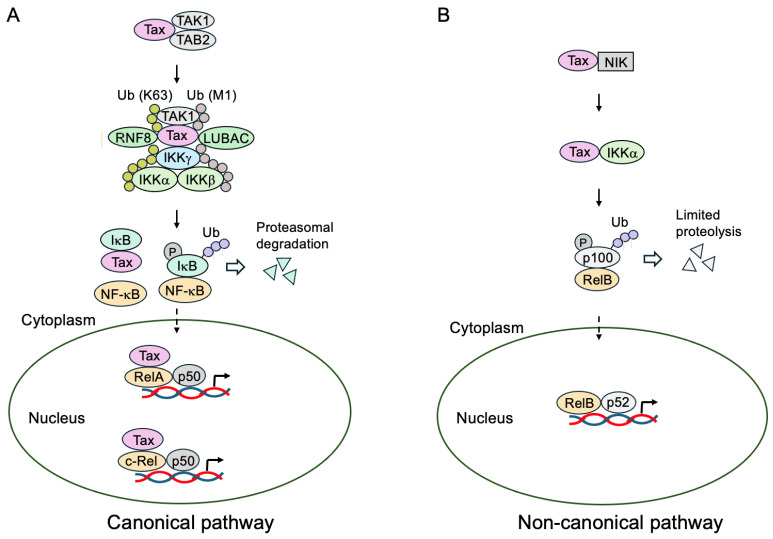
The mechanisms of activation of the canonical and non-canonical NF-κB pathways by Tax. (**A**) Tax activates IKK complex through association with IKKγ (NEMO) and TAK1. Tax also binds to RNF8 and LUBAC, generating polyubiquitin chains to facilitate association between TAK1 and IKK. Activated IKK complex phosphorylates IκB targeting it for proteasome-mediated degradation, thereby releasing NF-κB. Tax associates with IκB and releases NF-κB. Tax also associates with RelA and c-Rel to enhance transcription. (**B**) Tax associates with and activates IKKα to phosphorylate p100, triggering limited proteolysis and generating p52. Tax also associates with and activates NIK to activate IKKα.

Tax activates the NF-κB pathway through a variety of mechanisms [28,37]. One way Tax activates the canonical NF-κB pathway is through activation of the IKK complex, leading to the phosphorylation and degradation of IκB through the ubiquitin–proteasome pathway (Figure 5) [134,135,136]. This process involves a variety of mechanisms and is explained in detail later.

Tax binds to the ankyrin motifs of IκBα, which is required for interaction with NF-κB, and inhibits binding to or the release of NF-κB and facilitates the proteasome-mediated degradation of IκBα (Figure 5A) [137,138]. Tax also binds to IκBβ and facilitates its degradation [139]. In contrast to IκBα, which is induced by the activation of NF-κB as a negative feedback mechanism, IκBβ expression is not induced by Tax. Hence, in Tax-expressing cells, IκBβ expression is scarcely detected, resulting the activation of c-Rel [139]. Tax also binds to ankyrin motifs of IκBγ (an alternative transcript of the *p105* gene) and releases NF-κB p65 to activate transcription [140]. Tax binds to p105, precursor of p50, and also two subunits of the 20S proteasome, HsN3 and HC9, and facilitates the interaction between p105 and HC9, likely promoting the processing of p105 to p50 and accumulation of p65/p50 in the nucleus [141,142,143]. In Tax-expressing cells, expression of p100, the precursor of p52, and c-Rel are enhanced; consequently, RelB/p52 and c-Rel/p50 are the principal components of NF-κB activity [144,145].

Tax also binds to the Rel homology domain of p65 and c-Rel and enhances transcriptional activity (Figure 5A) [146,147]. Consistent with this, Tax co-localizes with p65/p50, CBP/p300, and RNA pol II [148,149].

Tax activates IKK through a variety of mechanisms. Tax activates IKKα and IKKβ through direct interaction with the NF-κB essential modulator (NEMO) (IKKγ), which was isolated by complementation cloning using a flat cell variant of HTLV-1 Tax-transformed rat fibroblasts unresponsive to all NF-κB-activating stimuli tested (Figure 5A) [150,151,152,153,154,155,156]. Consistent with this, somatic mutagenesis assays demonstrated that a Jurkat T-cell line lacking the expression of NEMO was compromised in the activation of NF-κB, and exogenous introduction of NEMO restores the activation of NF-κB by Tax in the mutant cells [155]. In addition, the Tax point mutant M22 (G137A and L138S), which lacks the ability to activate NF-κB, is also compromised in binding to NEMO [152]. Efficient activation of NF-κB also involves the phosphorylation of RelA/p65 at Ser529 and Ser536 by IKKα [157]. Tax also activates kinases upstream of IKK to facilitate its activation. Tax binds to TGF-β-activating kinase 1 (TAK1) and TAK1-binding protein 2 (TAB2) and stimulates TAK1 kinase activity (Figure 5A) [158,159]. Since Tax also binds to the IKK complex through NEMO, Tax recruits TAK1 to the IKK complex to stimulate its kinase activity [158]. In addition, Tax binds to and activates MAP/ERK kinase kinase 1 (MEKK1), which also facilitates the activation of IKKβ [160].

Upon T-cell activation, IKK complexes are transiently recruited to the plasma membrane-associated lipid raft microdomains for the activation of NF-κB in promoting T-cell proliferation [161]. Tax modulates the localization of the IKK complex from cytoplasm to Golgi-associated lipid raft microdomains dependent on the presence of NEMO [162,163], where Optineurin and Tax-binding protein 1 (TAXBP1) cooperate to activate NF-κB [164]. TANK-binding kinase 1 (TBK1) also co-localizes with the Tax and IKK complex in the lipid raft microdomains and plays a crucial role in the activation of NF-κB and Stat3 [165].

In addition to activating the IKK complex by direct interaction, Tax also facilitates IKK complex activation through the formation of polyubiquitin chains, which function as a platform to recruit the IKK complex and regulatory factors [166]. Tax undergoes a variety of post-translational modifications including Lysine 63 (K63)-linked polyubiquitination, which provides an important regulatory mechanism that promotes the Tax-mediated interaction with the IKK complex and activation of NF-κB [167]. The E3/E4 ubiquitin conjugation factor UBE4B interacts with Tax and induces the K48- and K63-linked polyubiquitination of Tax [168].

Tax stimulates the ubiquitin E3 ligase ring finger protein 8 (RNF8) to assemble lysine 63 (K63)-linked polyubiquitin chains to which NEMO and TAB2/3 bind, thereby recruiting IKK and TAK1 (Figure 5A). This recruitment to K63-linked polyubiquitin chains facilitates the activation of TAK1 by cross-autophosphorylation, leading to TAK1-mediated IKK phosphorylation and activation [169]. Tax also recruits the (Met1-linked) ubiquitin E3 ligase, linear ubiquitin chain assembly complex (LUBAC), to the IKK complex, generating Lys63- and Met1-linked hybrid polyubiquitin chains (Figure 5A). This hybrid-chain-dependent oligomerization of the IKK complex leads to trans-autophosphorylation-mediated IKK activation [170]. The possibility that Tax itself functions as a putative ubiquitin E3 ligase for the activation of IKK via synthesis of mixed-linkage polyubiquitin chains is also reported [171].

Tax maintains persistent NF-κB activation by a number of mechanisms such as counteracting negative regulators of the NF-κB pathway, cross-talk between canonical and non-canonical pathways, and the establishment of feed-forward signaling loops [28,30].

Tax also activates the non-canonical NF-κB pathway by multiple mechanisms (Figure 5B). Processing of the RelB heterodimeric partner p52 precursor p100 to p52 is enhanced in HTLV-1-transformed cells. Tax associates with p100 [172]. Tax not only activates IKKα and IKKβ but also activates NF-κB-inducing kinase (NIK) [136], which activates IKKα to phosphorylate p100 to induce processing to p52 (Figure 5B). Tax, via IKKγ, and NIK recruit IKKα to p100 to facilitate the phosphorylation of p100 on serine residues, leading to ubiquitination by the β-transducin repeat-containing protein (β-TrCP) ubiquitin ligase and subsequent processing of p100 to p52 mediated by the 26S proteasome (Figure 5B) [173,174]. Although β-TrCP is essential for NIK-induced p100 ubiquitination and processing [175], the Tax-induced processing of p100 is only partially dependent on β-TrCP [176].

### 3.3. SRF

Tax activates immediate early response genes such as *c-fos* through activation of the serum response factor (SRF), which binds the serum response elements (SRE, CArG box). Tax binds not only to NF-κB but also to SRF to activate transcription [146]. Tax also interacts with the ternary complex factor (TCF), which binds to Ets box sequences (GGAA/T), and TCF binding is necessary for the Tax-mediated activation of the SRE [177]. Tax binding to SRF also enhances its binding affinity for divergent SRE sequences [178].

## 4. Cellular Target Genes of Tax

Target genes of Tax identified to date, in addition to the viral LTR, include those coding for cytokines, their receptors, growth signal transducers, proto-oncogenes, transcription factors, cell cycle regulators, apoptosis-related genes, and cell attachment molecules. Early transformation of virus-infected cells seems to be mediated through the co-operation of a repertoire of target genes, which promote cell proliferation and cell survival.

JPX-9 is a subline of the human T-cell line Jurkat, in which expression of the *Tax* gene is driven by the metallothionein promoter, enabling the induction of Tax expression by the addition of heavy metal ions, such as Cd^2+^ and Zn^2+^, with little, if any, expression in their absence [179]. This cell line has been successfully utilized in many laboratories to identify the target genes of Tax and to examine the effects of Tax [180,181,182,183,184,185,186,187,188,189,190,191,192,193,194,195,196,197,198,199,200,201,202,203,204,205,206,207].

### 4.1. Cytokines, Their Receptors, and Cell Surface Molecules

Cytokines are low-molecular-weight proteins (5~20 kDa) secreted from a variety of cell types that include chemokines, interleukins (IL), interferons (IFN), lymphokines, and tumor necrosis factors (TNFs). The T-cell growth factor *IL-2* [208,209,210,211,212,213] and its receptor *IL-2Rα* [208,209,214,215,216,217,218,219,220] are among the first cellular target genes of Tax to be identified. The Tax-mediated induction of both genes is primarily mediated through the NF-κB pathway. Induction of both IL-2 and its receptor suggested autocrine growth stimulation as a possible mechanism underlying leukemogenesis induced by HTLV-1 Tax. However, IL-2Rα alone cannot transmit a growth signal into cells. IL-2R is composed of three components, IL-2Rα, IL-2Rβ, and IL-2Rγ. IL-2Rα contributes to the formation of a high affinity receptor, and IL-2Rβ and IL-2Rγ are required to transmit growth signals by coupling to the tyrosine kinases Janus kinase 1 (JAK1) and JAK3, respectively [221]. Tax also induces expression of the *IL-2Rγ* gene [222], suggesting the possibility that Tax increases the expression of the functional high affinity receptor for IL-2 [223]. ATL cell lines established from ATL patients are all dependent on IL-2 for their isolation and culture. However, IL-2-dependent ATL cell lines become IL-2-independent during further culturing [224]. These observations suggest the possible involvement of an IL-2/IL-2R autocrine mechanism in the early phase of transformation by HTLV-1 infection. In support of this notion, dual infection of the threadworm Strongyloides stercoralis (S. stercoralis) with HTLV-1 induces polyclonal expansion of the HTLV-1-infected cells by stimulating the IL-2/IL-2R system [225].

Tax also activates the *c-sis*/*platelet-derived growth factor-B* [226], *IL-9* [227,228], *IL-15*/*IL-15R* [229,230,231], and *IL-21*/*IL-21R* genes [232,233], which are involved in the activation, cell growth, and survival of T cells, suggesting that the Tax-mediated activation of these genes may also contribute to the proliferation of HTLV-1-infected T cells. The Tax-mediated activation of these genes is also mainly mediated through the NF-κB pathway [229,230,231,232].

HTLV-1-infected T-cell lines and fresh leukemic cells isolated from patients with ATL express high levels of *TGF-β1* mRNA and secrete TGF-β1. Tax activates the TGF-β1 promoter through activator protein 1 (AP-1) binding sites [234]. In addition, tumors from Tax transgenic mice, under the control of HTLV-1 LTR [72], express high levels of *TGF-β1* mRNA and protein. Moreover, TGF-β1 stimulated the growth of the tumor cells, suggesting that the Tax-mediated induction of TGF-β1 may play a role in tumorigenesis [235].

Tax activates the *gp34* gene [181], which codes for tumor necrosis factor ligand superfamily member 4, the ligands for OX40, a member of the TNF receptor family [236]. OX40 is expressed on activated T cells and is a costimulatory T-cell molecule known to be upregulated in HTLV-1-infected cells [189,237,238]. Tax activation of both genes is mediated through the NF-κB pathway [237,239]. The Tax-mediated induction of OX40 and its ligand gp34 may transmit growth signals and facilitate the proliferation of HTLV-1-infected cells [4,223]. Intriguingly, the Tax of HTLV-1 (Tax1) but not of HTLV-2 (Tax2), which is not associated with pathogenesis of ATL, induces OX40L expression through the activation of NF-κB2/p100/p52 [240]. Expression levels of OX40 are also associated with disease progression in patients with HAM/TSP [238].

Tax induces the aberrant expression of CD40, a member of the tumor necrosis factor receptor (TNFR) family that plays an important role in lymphocyte activation and differentiation [241]. Tax activates the *CD40* gene through the NF-κB signaling pathway. Since Tax also induces expression of the CD40 ligand (CD40L) and ligation of CD40 on T cells with recombinant CD40L elicited NF-κB activation, the CD40/CD40L pathway may form a positive regulatory loop in NF-κB activation in HTLV-1-infected T cells [242].

HTLV-1-transformed T cells and fresh ATL leukemic cells produce vascular endothelial growth factor (VEGF) and basic fibroblast growth factor (bFGF), which induce angiogenesis. Tax also activates the VEGF promoter [243].

ATL is often accompanied by hypercalcemia, and the expression of parathyroid hormone-related peptide (PTHrP) is increased in all the ATL patients examined [244]. PTHrP increases serum calcium concentration by the same mechanism as the parathyroid hormone (PTH). PTHrP expression is increased in asymptomatic carriers without hypercalcemia but is not elevated in HTLV-1-irrelevant leukemic cells [240]. Tax activates the *PTHrP* gene [245] and PTHrP P2 promoter through Ets1, AP1/c-jun, Sp1, and the NF-κB pathway [246,247,248,249]. Expression of the PTHrP receptor (PTH1R) is also increased in HTLV-1-infected lymphocytes, indicating a potential autocrine role for PTHrP [250]. However, whether PTHrP signaling facilitates the growth of HTLV-1-infected cells has yet to be elucidated.

Chemokines and their receptors play important roles in the migration and tissue localization of various lymphocyte subsets. C-C chemokine receptor type 4 (CCR4) is the receptor for CC chemokines CCL2 (MCP-1), CCL4 (MIP-1), CCL5 (RANTES), CCL17 (TARC), and CCL22 (macrophage-derived chemokine: MDC). CCR4 expression is enhanced in primary ATL cells and HTLV-1-immortalized T cells. Primary ATL cells show efficient migration in response to the CCR4 ligands CCL17/TARC and CCL22/MDC in chemotaxis assays. Since CCR4 is known to be involved in T-cell migration into skin, Tax-mediated CCR4 expression may explain the frequent dermal infiltration of ATL cells [251,252]. Tax induces CCR4 expression through the induction of Fos-related antigen 2 (Fra-2), which forms Fra-2/JunB and Fra-2/JunD heterodimers to activate an AP-1 site in the CCR4 promoter. Fra-2 also induces the expression of c-Myb, BCL-6, and MDM2, thereby promoting cell growth [253]. CXCR7 is an atypical chemokine receptor, frequently expressed by tumor cells, known to promote cell growth and survival. Tax induces the expression of CXCR7 through NF-κB and promotes the growth and survival of HTLV-1-infected T cells [186].

HTLV-1 infection causes not only ATL but also several inflammatory diseases such as HTLV-associated myelopathy/tropical spastic paraparesis (HAM/TSP). Tax activates a variety of genes involved in inflammation and immune response in addition to cell growth, such as *IL-1α* [254,255,256,257,258], *IL-1β* [256,259], *IL-4* [260], *IL-5* [203,227,261], *IL-6*/*IL-6R* [188,254,256,262,263,264,265,266], *IL-8* [184,205,267], *IL-9* [266], *IL-10* [83,268,269], *IL-12* [270], *IL-13* [227,271,272,273], *IL-17*/*IL-17R* [274,275], *IL-21*/*IL-21R* [232,233], *IFN-γ* [270,271,276], *TNF-α* [256,270,277,278,279], *TNF-β* or *lymphotoxin* [256,280,281], *granulocyte–macrophage colony-stimulating factor* (*GM-CSF*) or *colony-stimulating factor 2* (*CSF2*) [209,218,282,283], *granulocyte colony-stimulating factor* (*G-CSF*) [283], *monocyte chemoattractant protein-1* (*MCP-1*) [270,284,285], *MCP-3* [270], *C-C motif chemokine ligand 1* (*CCL1*) [286], *CCL3*/*macrophage inflammatory protein 1α* (*MIP-1α*) [184,284,285,287], CCR4 ligand *CCL4*/*MIP-1β* [288,289], CCR4 ligand *CCL5*/*regulated on activation, normal T cell expressed and secreted* (*RANTES*) [184,284,290], *CCL11*/*Eotaxin* [270], *CCL20*/*MIP-3α* and its receptor *CCR6* [291], CCR4 ligand *CCL22* [292], *chemokine, C motif, ligand 1* (*XCL1*, *SCM1*) [184], *chemokine, CXC motif, ligand 10* (*CXCL10*) (*IP-10*) [184,284,293,294], *CXCL12* (*SDF-1*, *PBSF*) [295], *C-C chemokine receptor type 9* (*CCR9*) [198], *C-X-C chemokine receptor type 4* (*CXCR4*) [296], and *CXCR7* [186]. CXCR7 is an atypical chemokine receptor frequently expressed by tumor cells and known to promote cell growth and survival. Tax induces the expression of CXCR7 through NF-κB and promotes the growth and survival of HTLV-1-infected T cells [186]. Most of these genes are activated by Tax through the NF-κB pathway. The Tax-mediated activation of these genes is likely to be involved in the onset of HTLV-1-associated pathogenesis such as inflammatory diseases and/or ATL. Consistent with this, the addition of IL-10 to Tax-depleted primary ATL cells rescues the survival of the Tax-depleted cells, suggesting that Tax-induced expression of IL-10 plays a critical role in the survival of HTLV-1-infected cells [83]. Expression of repulsive guidance molecule A (RGMa), which is known to contribute to neuronal damage, is upregulated in HTLV-1-infected cells from patients with HAM, and Tax induces the expression of RGMa [297]. Tax also enhances the expression of T-box transcription factor (T-bet) and induces the Th1-like state, consequently promoting the production of IFN-γ, which enhances inflammation [276]. Tax-induced RGMa and T-bet may also contribute to the pathogenesis of HAM.

HTLV-1-infected cells show an increased expression of vascular cell adhesion molecule 1 (VCAM-1, CD106), and Tax activates the VCAM-1 promoter through an NF-κB binding site [204]. Tax also induces the expression of leucocyte function-associated antigen-3 (LFA-3) [191]. Tax-induced VCAM-1 acts in synergy with leucocyte function-associated antigen-3 (LFA-3) to promote the interaction between T cells and increase T-cell proliferation [298].

Cell adhesion molecule 1 (CADM-1), also known as the tumor suppressor in lung cancer 1 (TSLC1) or immunoglobulin superfamily member 4 (IGSF4), is a cell adhesion molecule that is expressed in most cell types, except for peripheral blood lymphocytes, and is thought to function as a tumor suppressor in a variety of cancers [299,300]. Expression of CADM1 is enhanced in HTLV-1-infected T-cell lines and primary ATL cells, and the percentage of CADM1(+) cells in HTLV-1 carriers and ATL patients correlates well with viral loads, indicating that CADM1 serves as a diagnostic cell surface marker for ATL [301]. The roles of CADM1 in ATL pathogenesis have yet to be elucidated.

Osteopontin (OPN) is a ligand for CD44, which promotes cancer cell growth, invasion, and metastasis. CD44 associates with a variety of growth factor receptors and enhances ligand binding, thereby promoting cell proliferation. Tax induces the expression of both OPN and CD44 through AP-1 and non-canonical NF-κB pathways, respectively [302,303,304], suggesting the involvement of OPN/CD44 in the growth of HTLV-1-infected cells.

Tax induces the expression of CD137. CD137 is transferred by trogocytosis to CD137L-expressing APC, where the CD137-CD137L complex is internalized and degraded, resulting in a reduced CD137-mediated T-cell co-stimulation and a weakened cellular immune response, which may facilitate the escape of the virus from immune surveillance [305].

Other cell surface molecules reported to be upregulated by Tax include E-cadherin [306], CD44 [303], CD83 [193], tumor necrosis factor receptor superfamily member 9 (TNFRSF9/4–1BB/CD137/ILA) [307], tumor necrosis factor ligand superfamily member 7 (CD70) [308], early activation marker of lymphocytes CD69 [309], and major histocompatibility complex, class I [310].

Tax transgenic mice under the control of HLV-1 LTR develop mesenchymal tumors including neurofibroma [73]. Tumors and tumor cell lines derived from these mice produce nerve growth factors (NGFs), and Tax trans-activates the NGF promoter through a Tax-responsive element exhibiting a 92% homology to the 21 bp repeats of the HTLV-1 LTR. The NGF receptor is also expressed in transgenic tumor cells, suggesting that Tax may activate an autocrine mechanism through the upregulation of NGF [311].

Enkephalins are opioid peptides that function as neurotransmitters and neuroimmunomodulators. Tax activates the proenkephalin gene in glial cells and activates the proenkephalin promoter through the increased binding of c-Fos/c-Jun proteins to the AP-1 site [312,313,314].

Activation of cytokines, their receptors, and other cell surface molecules, which transmit growth signals, may facilitate the proliferation of HTLV-1-infected cells at early stages of expansion of the virus-infected cells.

The genes among those mentioned above, which have been reported to be induced by Tax, are summarized in Table 1.

### 4.2. Proto-Oncogenes, Transcription Factors, and Growth Signal Transducers

Tax activates a variety of proto-oncogenes and growth signal transducers, including early response genes such as *c-Fos* [180,315,316], *Fra-1* [206], *v-jun avian sarcoma virus 17 oncogene homolog* (*c-Jun*) [206], *JunD* [206], *early growth response 1* (*Egr-1*) [206,317,318], *Egr-2* [206,318], *ETR101* [202], *butyrate response factor 1* (*BRF1*) [319], and *c-Myc* [320]. Dimerization between the Jun (c-Jun, JunB, and JunD), Fos (c-Fos, FosB, Fra1, and Fra2), and activating transcription factor (ATF) protein families generate AP-1 transcription activity, which plays an important role in tumorigenesis [321]. Accordingly, primary ATL cells and HTLV-1-infected cells exhibit constitutively active AP-1 [322,323,324]. Tax activates the *Egr-1* and *Egr-2* genes through SRF [318]. Tax also activates the *Egr-1* gene through the NF-κB pathway and stabilizes the Egr-1 protein by direct interaction. Elevated Egr-1 in turn facilitates the translocation of p65 into the nucleus, leading to increased NF-κB activation [325]. These results suggest a positive feedback loop in Egr-1 activation and NF-κB activation in Tax-expressing cells.

Tax increases c-Rel and p105 mRNA expression [326], although it may not be directly mediated by Tax, suggesting that the induction of expression of these genes may also contribute to the activation of NF-κB by Tax. Tax-mediated induction of the *c-Rel* gene is mediated through the activation of an intrinsic κB enhancer element in the c-Rel promoter by RelA/p65 [134].

The Pim family of serine/threonine kinases (Pim-1, -2, and -3) promote tumorigenesis by enhancing cell survival. Tax induces the expression of Pim-1 and Pim-3 through NF-κB, which may contribute to the cell growth and survival of HTLV-1-infected cells [327,328]. Conversely, Pim-1 and Pim-3 bind to Tax and reduce its expression, thereby creating a negative feedback loop. Since Tax protein is highly immunogenic, Tax-mediated Pim-1 and Pim-3 induction may contribute to the escape of HTLV-1-infected cells from immune surveillance by reducing Tax protein expression [328]. Pim kinases may serve as a target for the treatment of ATL [329].

The signal transducer and activator of transcription (STAT) is a family of transcription factors which transmit cytokine signals from membrane receptors coupled with JAK kinases into the nucleus. The activation of the JAK-STAT pathway and the expression of *STAT1*, *STAT3*, and *STAT5* mRNAs are induced by mitogen stimulation of normal human peripheral blood mononuclear cells. In contrast, HTLV-1-infected T cells show constitutive activation of the JAK-STAT pathway [330,331]. In addition, HTLV-1-infected T-cell lines also show an enhanced expression of *STAT1*, *STAT3*, and *STAT5* genes, and Tax induces the expression of *STAT1* and *STAT5* genes in a human T-cell line [332]. IL-2 stimulation enhanced the DNA-binding activity of STAT3 and STAT5 in a HTLV-1-transformed cell line concomitant with its proliferation. The induction of STAT1 and STAT5 by Tax may enhance cytokine-induced proliferation of the virus-infected T cells, thereby playing a role in the IL-2-dependent transformation of T cells in response to HTLV-1 infection.

Interferon regulatory factor (IRF-4) was shown to be constitutively produced in HTLV-1-infected cells but not in continuously growing non-Tax-expressing T-cell lines. In contrast to other IRFs, IRF-4 is not induced by INFs but is transiently expressed upon growth stimulation of T lymphocytes, such as anti-CD3 and phorbol 12-myristate 13-acetate (PMA)/ionomycin stimulation. Tax activates the IRF-4 promoter through the NF-κB and ATF pathway, suggesting that IRF4 induction in HTLV-1-infected cells is mediated through Tax activation of the IRF-4 gene transcription [333,334]. Intriguingly, an activating mutation of IRF4 is frequently observed in ATL [335]. IRF5 is also constitutively expressed in HTLV-1-infected T cells and Tax activates the IRF5 promoter [336]. Tax can indirectly activate the interferon-responsive enhancer element of the *2′,5′-oligoadenylate synthetase* gene in an NF-κB pathway-dependent manner [337].

The Src family of protein-tyrosine kinases (Lck, Fyn, and Lyn) is associated with growth signal transduction, including cytokine signaling. Expression of the *Lyn* gene is detected in HTLV-1-infected and Tax-expressing T-cell lines but not in normal T cells [338]. Tax induces expression of the *Lyn* gene and activates the Lyn promoter in T cells, suggesting that the induction of Lyn gene transcription in HTLV-1-infected cells is mediated through Tax [339]. Lyn is associated with IL-2Rβ and JAK3, and dominant negative Lyn reduced the phosphorylation of JAK3 and STAT5, suggesting the involvement of Lyn in IL-2R signaling. These results suggest that the induction of Lyn expression by Tax may facilitate IL-2R signaling, although Lyn is not essential for IL-2R signal transduction [340].

Expression of key genes associated with T-cell activation was compared between 20 asymptomatic carriers (ACs) of HTLV-1 and 20 healthy control subjects using RT-qPCR [341]. Expression (mRNA) of *glycogen synthase kinase-3β* (*GSK3β*), *mitogen-activated protein kinase kinase kinase 14* (*MAP3K14* or *NIK*), *phospholipase C γ 1* (*PLCG1*), and *mitogen-activated protein kinase kinase kinase-7* (*MAP3K7* or *TAK1*) were significantly higher in asymptomatic carriers (ACs) compared to healthy subjects, suggesting the involvement of these genes in the early stage of ATL development.

Activation of these early response and signal transducer genes may transmit growth signals in the absence of growth simulation, thereby contributing to the growth of HTLV-1-infected T cells.

Nur77 is a macrophage-expressed nuclear receptor that regulates the inflammatory response [342]. HTLV-1-infected T cells showed a higher expression of Nur77, and Tax induces Nur77 expression in JPX-9 cells [196]. Promoter analysis shows that the induction is mediated by CREB/ATF-related transcription factors [196,201]. Induction of Nur77 expression may be relevant in HTLV-1-related inflammatory diseases.

Expression of the Ellis Van Creveld (EVC), a positive mediator for three hedgehog (Hh) signaling molecules Desert (DHH), Indian (IHH), and Sonic (SHH), is upregulated in ATL and HTLV-1-infected cells. The downregulation of EVC leads to apoptotic cell death in ATL cell lines, indicating that HH activation is involved in the regulation of leukemic cell survival [343].

An inducibly expressed atypical IκB protein NF-κB-ζ (IκB-ζ) associates with the NF-κB subunit in the nucleus and positively regulates its transcriptional activity [344]. IκB-ζ is constitutively expressed in HTLV-1-infected T-cell lines and ATL cells, and Tax trans-activates the *IκB-ζ* gene, mainly through NF-κB [345]. The over-expression of IκB-ζ induces the expression of NF-κB and interferon-regulatory genes, such as *B cell CLL/lymphoma 3* (*BCL3*), *guanylate-binding protein 1*, and *signal transducer and activator of transcription 1* (*STAT1*) [345]. Interestingly, IκB-ζ interacts with Tax and inhibits the Tax-mediated activation of NF-κB, AP-1, and HTLV-1 transcription [345], suggesting that IκB-ζ may be involved in the pathogenesis of HTLV-1-associated diseases.

The comparison of gene expression profiles between 10 ATL patients, 10 ACs, and 10 normal controls revealed that Evi-1 and Foxo-1 levels were significantly higher in ATL patients [346]. Evi-1 and Foxo-1 are important factors in maintaining hematopoietic and embryonic stem cells, suggesting a role for both in HTLV-1-mediated transformation.

B-cell/CLL lymphoma 6 (BCL6) expression is enhanced in HTLV-1-infected T-cell lines, and Tax induces BCL6 expression. Knockdown or pharmacological inhibition of BCL6 reduced the cell growth and survival of HTLV-1-infected T-cell lines. BCL6 might be considered a novel target for ATL treatment [347].

Expression of Exportin 1 (XPO1), also known as chromosomal region maintenance 1 (CRM1), is upregulated in HTLV-1-infected T cells, and the knockdown of XPO1 reduced cell proliferation. The XPO1 inhibitor KPT-330 also reduced proliferation, increased DNA damage, and induced G1 cell cycle arrest and caspase-dependent apoptosis, suggesting the use of KPT-330 in clinical trials targeting ATL [348].

Expression of OSBP-related protein 4 (ORP4L), also known as oxysterol-binding protein 2 (OSBP2), is upregulated in ATL cells due to the loss of miR-31 expression caused by HTLV-1 infection. ORP4L knockout blocked T-cell leukemogenesis in Tax transgenic mouse models, whereas ORP4L expression in T cells resulted in T-cell leukemia in mice. ORP4L interacts with PI3Kδ to promote PI(3,4,5)P3 generation, thereby contributing to the activation of AKT, NF-κB, and T-cell leukemogenesis. Consistent with this, ORP4L knockout or pharmacological inhibition reduces the growth of ATL cells in patient-derived xenograft ATL models. These results point to ORP4L as a therapeutic target of ATL [349].

Notch signaling plays an important role during neuronal development. Notch is a cell surface protein and, upon binding of the ligand, such as Jagged (JAG1), the Notch intra-cellular domain (NICD) is cleaved and translocates into the nucleus to regulate gene expression. Notch signaling is constitutively activated in ATL cells, as determined by gene array analysis [350]. The Notch ligand, JAG1, is overexpressed in primary ATL cells compared to normal peripheral blood mononuclear cells (PBMCs). Tax induces JAG1 through the NF-κB pathway. Inhibition of JAG1 by knockdown or antibodies reduces Notch1 downstream signaling and limits the cell migration of transformed ATL cells [351], suggesting that JAG1 can be a new target to dampen Notch1 signaling in HTLV-1-transformed cells. F-box and WD repeat domain containing 7 (Fbw7), a component of E3 ubiquitin ligase, recognizes NICD and facilitates its degradation. Intriguingly, more than 30% of ATL patients possess activating mutations in Notch including those leading to reduced Fbw7-mediated degradation and the stabilization of NICD [352]. In addition, 25% of ATL patients possess mutations in Fbw7, which eliminates its interaction with NICD, resulting in increased protein stability and constitutive Notch1 signaling [353]. Notch signaling is activated in ATL cells irrespective of the presence of activating mutations in Notch itself or its repressor Fbw7, and lncRNA H19/miR-675 is highly expressed in ATL cells, contributing to Notch signaling activation [351]. In addition, Tax directly binds to Fbw7 in the nucleus and competes with its substrate to stabilize NICD [354]. Tax also increases NICD expression by stabilizing the half-life of NICD mRNA. Tax interacts with NICD and recombination signal Jκ binding protein (RBP-Jκ) and stabilizes binding of the ternary complex to the RBP-Jκ target sequence, thereby enhancing Notch signaling [355]. Inhibition of Notch signaling by knockdown or γ-secretase inhibitors reduces tumor cell proliferation and tumor formation in an ATL-xenograft mouse model [350,352,355], suggesting that the Tax-mediated enhancement of Notch signaling plays an important role in the proliferation of HTLV-1-infected cells.

HTLV-1 Tax induces and associates with Crk-associated substrate lymphocyte-type (Cas-L). Cas-L is a docking protein that is heavily tyrosine phosphorylated by the engagement of Integrin β1 in T cells. Fresh ATL cells and HTLV-1-infected T-cell lines show an elevated level of Cas-L. Tax induces expression and tyrosine phosphorylation of Cas-L in the Jurkat human T-cell line, resulting in increased motile behavior and response to integrins [185]. Tax also associates with Cas-L and exogenous Cas-L-inhibited Tax-mediated activation of NF-κB, suggesting that Cas-L might regulate Tax-mediated NF-κB activation [185]. Tax transgenic mice spontaneously develop rheumatoid arthritis (RA). Splenocytes from Tax transgenic mice show higher migratory activity with enhanced expression and phosphorylation of Cas-L than non-transgenic control [356]. In addition, in a mouse model of collagen-induced arthritis, Cas-L (−/−) mice showed a reduced severity of arthritis as compared to Cas-L (+/+) mice with a reduced immune response to collagen [357]. These observations suggest the involvement of Cas-L in the pathogenesis of RA.

Tax induces the expression of membrane-associated guanylate kinase, WW and PDZ domains-containing 3 (MAGI3) in Rat-1 cells [358]. MAGI3 has multiple PDZ domains and interacts with Tax in 293T cells, dependent on a PDZ domain-binding motif (PBM). PBM is not present in the Tax2 of HTLV-2 and is thought to be important for transforming the activity of Tax1 [359]. The interaction of Tax1 with MAGI-3 alters their respective subcellular localization and correlates well with the transforming activity of Tax, suggesting that the interaction of Tax with MAGI3 may play a role in the pathogenesis of HTLV-1-associated diseases [358]. Tax1 PBM also binds other PDZ domain-containing proteins such as Dlg1, Scribble, and MAGI-1 [360,361,362] and plays an important role in the IL-2 independent growth induction of a T-cell line [363], sustained T-cell proliferation in HTLV-1-infected humanized mice [364], and Akt pathway activation [365].

Expression of the *calpain 2* gene is increased in HTLV-1-infected cells, and the introduction of the *pX* gene into an NK-like cell line induced *calpain 2* gene expression, suggesting that Tax activates the *calpain 2* gene [366]. Calpain 2 localizes in lipid rafts in T cells, suggesting the involvement of calpain 2 in TCR signaling [367].

The genes among those mentioned above, which have been reported to be induced by Tax, are summarized in Table 2.

### 4.3. Cell Cycle Regulators

Cell cycle progression is governed by cyclin-dependent kinases (CDKs), whose activity is regulated by the expression of partner cyclins [368]. Growth stimulation induces the expression of D-type cyclins, which bind to and activate CDK4 and CDK6 [369,370,371]. CDK4 and CDK6 in turn phosphorylate and inactivate p130, a member of the pRB protein family, which is bound to repressor-type E2F (E2F4 and E2F5) to suppress growth-related E2F target genes [370,371,372]. This relieves repression and induces the expression of E2F targets including cyclin E and activator-type E2F (E2F1, E2F2, and E2F3a). Cyclin E binds to and activates CDK2, which further phosphorylates and inactivates pRB family members to enhance E2F activity [370,371,372]. Induced activator E2Fs replace repressor-type E2F to activate target genes. This enables passage through the restriction point in the late G1 phase and entry into the S phase [370,371,372]. Tax activates a variety of cell cycle regulatory genes to promote cell cycle progression.

Kit 225 is a human T-cell line whose growth is dependent on IL-2 and can be growth arrested by starvation of the IL-2 without significant cell death. This enables the examination of effects of Tax on cell cycle progression. In contrast, the growth of Jurkat human T cells is independent of IL-2 and does not enter a resting state in response to IL-2 deprivation. Tax can induce cell cycle progression in IL-2-starved Kit 225 cells with concomitant induction of cyclin D2 and the activation of E2F [71]. Tax can also stimulate cell cycle progression in phytohemagglutinin (PHA)-stimulated peripheral blood lymphocytes (PBLs), indicating that Tax can induce cell cycle progression in human normal T cells. Intriguingly, the Tax-mediated cell cycle progression of PHA-PBLs is observed in Tax-expressing cells but not in Tax-negative cells [71], suggesting that the cell cycle progression induced by Tax is a cell intrinsic event and not mediated through humoral factors under these experimental conditions. The expression of Tax in IL-2-starved Kit 225 cells increased the expression of cyclin D2, cyclin E, E2F1, CDK2, CDK4, and CDK6 and reduced that of the CDK inhibitors p19^INK4d^ and p27^Kip1^ [373]. The increased expression and activity of cyclin D2, CDK4, and CDK6 leads to the enhanced phosphorylation of RB family proteins, activation of E2F, and cell cycle progression [373]. The Tax-mediated induction of cyclin D1, cyclin D2, and E2F1 have also been reported in other experimental settings. HTLV-1-infected T-cell lines and ATL cells show the enhanced expression of cyclin D2, and Tax induces *cyclin D2* gene expression [374]. Introduction of Tax into the IL-2-dependent murine T-cell line CTLL-2 enables IL-2-independent growth with the concomitant induction of cyclin D1 and cyclin D2 [375], and the introduction of Tax into CEM human T cells, which lack the expression of p16^INK4a^, increased E2F1 expression [376]. Tax-mediated induction of cyclin D2 and CDK6 expression is mediated through the activation of transcription through NF-κB-like sequences in their promoters and is dependent on the NF-κB pathway [377,378]. Tax-mediated activation of the *cyclin D1* gene is mediated through CRE in co-operation with CREB, the transducer of regulated CREB 2 (TORC2) and p300 [117]. Intriguingly, Tax cannot activate E2F or induce cell cycle progression in fibroblasts, in which the over-expression of E2F1 could induce cell cycle progression [71], suggesting that the Tax-mediated activation of E2F and cell cycle progression are cell-type-specific effects of Tax. Knockdown of cyclin D2 and CDK6 suppresses cell cycle progression induced by Tax in Kit 225 cells. Tax cannot induce cyclin D2 or CDK6 expression or activate the cyclin D2 or CDK6 promoters in fibroblasts, and over-expression of cyclin D2 and CDK6 induces cell cycle progression in fibroblasts [378]. These results suggest that cell-type-specific activation of the *cyclin D2* and *CDK6* genes through the NF-κB pathway is important for Tax-mediated cell cycle progression.

In addition to the induction of partner cyclins, the activation of CDKs requires phosphorylation at the activation segment T-loop by CDK-activating kinase (CAK) [379]. CAK is composed of CDK7, Cyclin H, and Menage a trois homolog 1 (Mat1) [380,381], among which CDK7 is the catalytic component. Accordingly, CDK7 plays an essential role in cell cycle progression by activating other CDKs (CDK1, 2, 4, and 6) involved in cell cycle progression. CAK, with its catalytic component CDK7, is also a component of transcription factor II H (TFIIH), which is required for the initiation of transcription by RNA polymerase II. RNA polymerase II recruited to target genes is paused at the transcriptional start site by anchoring of the C-terminal domain (CTD) of RNA polymerase II. To initiate transcription, RNA polymerase II needs to be released from anchoring by phosphorylation of the CTD catalyzed by CDK7 in TFIIH [382,383]. Accordingly, CDK7 plays an essential role in the initiation of transcription by RNA polymerase II. Tax induces the expression of CDK7 but not its partner Cyclin H [384]. In spite of this, Tax enhances phosphorylation of CDK2 and the CTD of RNA polymerase II [384], which are reported to be mediated by CDK7. Moreover, the knockdown of CDK7 suppressed the induction of target gene expression and cell cycle progression induced by Tax [384]. These observations suggest a unique relationship between CDK7 and Cyclin H, opposite to that of other CDKs and cyclins in the control of its activity. Since a basic mechanism of Tax-mediated promotion of cell cycle progression is the activation of target genes and CDKs, the *Cdk7* gene could be a crucial target of Tax in the proliferation of HTLV-1-infected cells.

RNA polymerase II released from transcription initiation sites still pauses at a promoter proximal site [385]. This pause is released by the function of the positive transcription elongation factor (P-TEFb), which contains Cdk9/cyclin T as a catalytic component. Tax induces the expression of the transcription elongation factor for RNA polymerase II 2 (ELL2) in HTLV-1-infected T cells [386]. Tax also interacts with ELL2 and synergistically activates the HTLV-1 LTR [386,387]. These results suggest that Tax also facilitates the elongation of transcription by modulating RNA polymerase II progression.

Primary ATL cells and Tax-expressing cells exhibit higher amounts of cyclin-dependent kinase inhibitor 1A (CDKN1A, p21^Cip1^) mRNA and protein compared to Tax-negative cells [388,389,390]. This is likely due to activation of the *CDKN1A* gene by Tax, since Tax activates the p21^Cip1^ promoter through an E2A transcription factor binding site, the CREB/ATF pathway, and two TGF-β-inducible Sp1 binding sites, independently of p53 [389,391,392]. Although p21^Cip1^ binds to and inhibits the CDK2/cyclin complex, it facilitates the assembly of cyclin D and CDK4 or CDK6 and enhances kinase activity [393,394]. Indeed, in HTLV-1-infected cells, p21^Cip1^ is in a complex with cyclin D2/CDK4, which retains kinase activity to phosphorylate pRB [395]. Although Tax induces the expression of p21^Cip1^, p21^Cip1^ is phosphorylated through the Tax-mediated activation of PI3/AKT and retained in the cytoplasm in an inactive state. In addition, in ATL cells, the expression of p21 is not increased, likely due in part to methylation of the gene [396].

Tax also promotes cell cycle progression by directly interacting with cell cycle regulators. Tax associates with cyclin D3/CDK6 [397]. Tax binds to and activates CDK4, thereby facilitating the phosphorylation of pRB [398]. Tax also associates with pRB and facilitates its proteasomal degradation [399]. Tax binds to and inactivates CDK inhibitors p16^INK4a^ and p15^INK4b^, leading to the activation of CDK4 [400,401,402].

Although Tax facilitates progression through the G1 and S phase transition [403], Tax delays progression through S-G2-M by directly binding to and prematurely activating the Cdc20-associated anaphase-promoting complex (APC/C^Cdc20^), an E3 ubiquitin ligase that controls the metaphase to anaphase transition [404,405]. This leads to premature degradation of cyclin B in S-G2 phases and may explain the mitotic abnormalities observed in HTLV-1-infected cells.

The genes among those mentioned above, which have been reported to be induced by Tax, are summarized in Table 3.

### 4.4. Genes Related to Apoptosis and Cell Survival

Apoptosis is induced by a cascade of activation of caspases which degrade cellular components, including DNA by activating DNase. The activation of caspase 9, an initiator caspase, is initiated by the release of cytochrome *c* from the mitochondria by destabilization of the mitochondrial outer membrane. Cytochrome *c* in turn forms the apoptosome, together with apoptotic protease-activating factor 1 (Apaf-1), in the cytosol to activate caspase 9. Caspase 9 then activates caspase 3 to initiate a cascade of activation of caspases 3, 6, and 7. Accordingly, stability of the outer membrane of the mitochondria is a key determinant of cell survival versus the induction of apoptosis. Anti-apoptotic Bcl-2 family proteins stabilize the outer membrane of the mitochondria and promote cell survival, while pro-apoptotic Bcl-2 family proteins destabilize it to promote apoptosis. Death receptors such as Fas activate caspase 8, an initiator caspase, upon the binding of a death ligand, such as the Fas ligand, to activate caspase 3 to initiate the caspase activation cascade.

Tax not only facilitates cell cycle progression but also cell survival by activating a variety of anti-apoptotic genes, indicated by changes in the gene expression profiles of HTLV-1-immortalized T cells, determined by DNA micro array, compared to normal T cells [406]. Many of the differentially expressed genes identified in HTLV-1-immortalized T cells are involved in the control of apoptosis, suggesting that deregulation of the apoptotic signaling network may play a role in the HTLV-1-mediated oncogenesis. Activation of anti-apoptotic genes by Tax is also mainly mediated through the NF-κB pathway.

One way Tax suppresses apoptosis is through stabilization of the mitochondrial outer membrane through the induction of anti-apoptotic Bcl-2 family members. Expression of Tax, in the IL-2-dependent murine T-cell line CTLL-2, alleviates apoptosis induced by deprivation of IL-2 concomitant with the induction of Bcl-xL expression via the NF-κB pathway [407,408]. Consistent with this, the Bcl-2/Bcl-xL inhibitor navitoclax in combination with the JAK inhibitor ruxolitinib reduces the IL-2-dependent ex vivo proliferation of peripheral blood mononuclear cells (PBMCs) from ATL patients in smoldering/chronic stages [409]. Induction of Tax in JPX-9 cells also suppresses receptor-mediated apoptosis induced by the anti-Fas antibody and tumor necrosis factor-related apoptosis-inducing ligand (TRAIL) and inhibits chemical-induced apoptosis (pyrrolidine dithiocarbamate, etoposide, and staurosporine) [182]. Tax-mediated induction of Bcl2-related protein A1 (BFL1) through the NF-κB pathway also contributes to cell survival [410].

Tax also suppresses apoptosis by inhibiting the activation of caspases. Inhibitors of apoptosis (IAPs) act to block the activation of caspases. Expression of baculoviral IAP repeat-containing 3 (BIRC3, cIAP2, and HIAP-1) is upregulated in HTLV-1-infected T-cell lines and ATL cells, and the induction of cIAP2 by Tax is mediated through NF-κB. Knockdown of cIAP2 increased apoptosis of HTLV-1-infected cells [411]. Expression of cIAP2 is also induced in HTLV-1 infected CD8(+) T cells through the NF-κB pathway, rendering the cells resistant to Fas-mediated cell death [412]. The Tax-mediated induction of X-chromosome-linked inhibitor of apoptosis (XIAP) through NF-κB also contributes to cell survival [183]. Survivin belongs to the IAP family and suppresses the activation of caspases. Expression of survivin is increased in HTLV-1-infected T-cell lines and primary ATL cells, with levels correlating with ATL prognosis [413,414]. Introduction of Tax into the IL-2-dependent T-cell line CTLL-2 induces the expression of survivin and protects cells from apoptosis, conferring IL-2-independent growth [415]. Tax increases the expression of cellular FLICE-inhibitory protein (c-FLIP), which binds to and inhibits Fas-associated proteins with the death domain (FADD) through NF-κB, thereby suppressing Fas-mediated apoptosis [416,417,418].

Ubiquitin-editing enzyme A20 not only suppresses the activation of NF-κB but also suppresses apoptosis by inhibiting caspase activation. In endothelial cells, A20 suppresses apoptosis induced by TNF, Fas, and natural killer cells by inhibiting the activation of caspase 8 [419]. Tax induces the expression of A20 through NF-κB in Jurkat T cells [420], and A20 is abundantly expressed in primary ATL cells [421]. A20 stably interacts with caspase 8 and FADD in HTLV-1-infected cells and suppresses caspase activation and apoptosis independent of its ubiquitin-editing catalytic activity [421].

Tax also facilitates cell survival through protein interaction. Tax induces lysine 63 (K63)-linked polyubiquitination of the myeloid cell leukemia sequence 1 (Mcl-1), an anti-apoptotic Bcl-2 family member, depending on TNF receptor-associated factor 6 (TRAF6). This stabilizes the Mcl-1 protein, thereby playing an important role in the survival of HTLV-1-transformed cells [422].

Multidrug resistance protein 1 (MDR1), also known as ATP-binding cassette sub-family B member 1 (ABCB1) or permeability glycoprotein (P-glycoprotein, P-gp), is a membrane glycoprotein that pumps many foreign substances out of cells, including anticancer drugs, thereby playing an important role in chemoresistance. HTLV-1-infected cell lines and primary ATL cells show enhanced MDR1 expression, and Tax activates the MDR1 promoter through an NF-IL6-binding site [423,424,425], suggesting the possibility that Tax-mediated induction of MDR1 may play a role in the drug resistance of ATL cells. Expression of Tax in S1T cells, a leukemic non-Tax-producing T-cell clone established from an ATL patient, conferred resistance to doxorubicin, etoposide, and vindesine but not to cisplatin. Expression of the *lung resistance-related protein* (*LRP*) gene was increased, but those of *MDR1*, *multidrug resistance-associated protein 1* (*MRP1*), and *multi-specific organic anion transporter* and *canalicular* (*cMOAT*) genes were not significantly changed [426], suggesting that Tax-mediated drug resistance of ATL cells is due to LRP and not MDR1 in this context.

Activation of these anti-apoptotic genes by Tax, mainly through the NF-κB pathway, and the enhancement of their activity are thought to play crucial roles in the survival of HTLV-1-infected cells. ATL is known to be extremely resistant to chemotherapy, which may be due, at least in part, to the Tax-mediated promotion of cell survival.

Intriguingly, Tax can also activate some pro-apoptotic genes and facilitate apoptosis under some experimental conditions. Expression of Tax in Jurkat T cells induces significant apoptosis concomitant with increased *Fas ligand* (*FasL*) and *TNF-related apoptosis-inducing ligand* (*TRAIL*) gene expression [190,427,428]. Tax activates the FasL promoter through the CREB and NF-κB pathways [190] and the *TRAIL* gene via NF-κB [428].

The genes among those mentioned above, which have been reported to be induced by Tax, are summarized in Table 4.

### 4.5. Human Telomerase Reverse Transcriptase (hTERT)

Most somatic cells have a finite replicative life span (40~50 cell divisions) determined by the length of telomere DNA, which is shortened every cell division due to the end replication problem. Hence, the expression of human telomerase reverse transcriptase (hTERT) is required for the immortalization of cancer cells to maintain telomere length.

Although initial evidence suggested Tax represses hTERT promoter activity [429], later studies indicate that Tax activates the *hTERT* gene. Infection of primary T cells with HTLV-1 induces hTERT expression, and Tax activates the hTERT promoter through the NF-κB pathway [430]. Intriguingly, T cells are unique among somatic cells in that growth stimulation, such as treatment with IL-2, induces the expression of hTERT [431]. Tax also induces expression of the *hTERT* gene in human peripheral blood leukocytes. Interestingly, *hTERT* gene expression is tightly linked with progression from the G1 to S phase of the cell cycle, suggesting that it is regulated by transcription factor(s) associated with the G1 to S phase transition [432]. Consistent with this, in HTLV-1-positive CD4(+) cells cloned from patients, hTERT expression parallels Tax expression and cell cycling [433]. Transcription-independent activation of hTERT enzymatic activity by Tax is also suggested to be mediated through the activation of the NF-κB pathway [434]. Induction of hTERT by Tax is thought to be important for the immortalization of HTLV-1-infected T cells.

### 4.6. Genes Involved in Suppression of DNA Repair and Induction of Genome Instability

Although Tax plays a crucial role in the early-stage transformation of HTLV-1-infected T cells, malignant transformation of the virus-infected cells is thought to require additional genetic changes. Indeed, ATL cells possess a variety of gene mutations involved in growth signal transduction, the maintenance of cell survival, and other pathways [59,60,61]. Tax increases the frequency of cellular DNA mutations and causes genomic instability by the induction of target gene expression and protein interactions to generate mutations required for malignant transformation of the virus-infected cells [435]. Tax-expressing cells show defective DNA repair, especially base-excision repair of oxidative damage [436], and are more likely to undergo gene amplification than control cells [437]. Tax also increases genetic instability by inducing DNA double strand breaks (DDSBs) during DNA replication and switches DNA repair from homologous recombination (HR) to the non-homologous end-joining (NHEJ) pathway, which is error prone, through activation of the NF-κB pathway [438]. These effects of Tax increase genetic and genome instability, which may be responsible for the accumulation of mutations, leading to malignant transformation [63].

Tax activates the *proliferating cell nuclear antigen* (*PCNA*) gene and increases PCNA protein expression [439,440,441], likely by relieving repression of the PCNA promoter [442]. Tax-expressing cells show a reduced capacity for nucleotide excision repair (NER), which correlates with the ability of Tax to activate the *PCNA* gene. Moreover, over-expression of PCNA reduces NER [440,441]. These observations suggest that Tax-mediated over-expression of PCNA reduces NER and predisposes cells to accumulate mutations, leading to the transformation of HTLV-1-infected cells.

Nitric oxide synthase (NOS) catalyzes the production of nitric oxide (NO). The *inducible nitric oxide synthase* (*iNOS*) gene is over-expressed in HTLV-1-infected and Tax-expressing cells, and Tax activates the iNOS promoter [443,444]. Tax induces iNOS expression in the human monoblast cell line, U937 [445]. Tax expression causes DNA double strand breaks (DSBs), concomitant with increased levels of NO and the induction of iNOS. Conversely, the inhibition of NO production dramatically reduces DSBs [446,447]. Taken together, these observations suggest that Tax promotes DSBs through, at least in part, activation of the *iNOS* gene. Tax also induces DNA damage by increasing reactive oxygen species (ROS) [448,449] and promotes cell cycle progression in the presence of DNA damage by suppressing checkpoints, thereby increasing genetic instability [450].

R-loops are structures consisting of a duplex of transcribed RNA and template DNA and an unpaired non-templated DNA strand. R-loops can be formed by invasion of nascent RNA into the DNA duplex to displace the non-template strand. Hence, highly transcribed regions tend to form R-loops. R-loop excision by nucleotide excision repair (NER) promotes DNA double-strand breaks (DSBs). The Tax-mediated constitutive activation of NF-κB increases R-loop accumulation and DNA double-strand breaks. Accordingly, defects in nucleotide excision repair are selected in adult T-cell leukemia [451,452].

Tax induces and activates WIP1 phosphatase, which dephosphorylates γH2AX and marks the location of damaged DNA, thereby mitigating the cellular response to DNA damage [453]. Consistent with this, Tax+Wip1−/− mice show a statistically significant reduced prevalence of tumorigenesis compared to their Tax+Wip1+/+ counterparts [454].

Tax also facilitates genome instability by protein interaction. Tax interacts with checkpoint kinase 2 (Chk2) and co-localizes with Chk2 and p53-binding protein 1 (p53BP1) [455]. Tax also interacts with ataxia-telangiectasia mutated (ATM), Chk1, and Chk2 and attenuates the DNA damage response [456,457,458]. Tax associates with minichromosome maintenance proteins (MCMs) and accelerates the replication timing, thereby generating replicative stress [459]. Tax binds to and inhibits topoisomerase I, suggesting the possibility that Tax affects cellular processes such as the transcription and maintenance of genomic stability in which DNA topoisomerase I is involved [460,461]. Tax forms microscopically visible nuclear speckles, termed Tax-speckle structures (TSS), depending on the activity of RNF8, which sequesters DNA damage response factors such as breast cancer 1 (BRCA1), DNA-dependent protein kinase (DNA-PK), and mediator of DNA damage checkpoint protein 1 (MDC1), thereby compromising DSB repair [462].

The genes among those mentioned above, which have been reported to be induced by Tax, are summarized in Table 5.

### 4.7. MicroRNAs (miRNAs)

MicroRNAs (miRNAs) are short non-coding RNAs that bind complementary mRNAs and silence gene expression by RNA interference. Transcribed precursor miRNAs are processed by ribonucleases DROSHA and DICER to generate mature miRNAs and are assembled into RNA-induced silencing complexes (RISCs) with Argonaute proteins. RISCs bind to mRNAs through complementary miRNA and degrade or inhibit the translation of target mRNAs [463,464,465,466]. Since the length of miRNAs is relatively short (around 22 base), a miRNA can affect multiple targets with similar sequences and effect their growth-promoting and tumor-suppressive functions. miRNAs targeting growth-promoting genes can function as tumor suppressors, while those targeting tumor suppressive genes can function as onco-miRNA. Several miRNAs are reported to be deregulated in HTLV-1-infected and ATL cells, and the involvement of miRNA deregulation in HTLV-1-mediated transformation has been suggested [29,467].

Several miRNAs including miR-34a, miR-146a, miR-155, miR-93, and miR-130b are upregulated in HTLV-1-infected T cells, mostly through the NF-κB pathway. miR-34a is a direct target gene of the tumor suppressor p53 and ectopic miR-34a expression induces apoptosis, cell cycle arrest, or senescence. Hence miR-34a expression is suppressed in many types of cancers [468,469,470]. Intriguingly, however, miR-34a expression is increased in HTLV-1-infected cell lines and ATL cells compared to normal CD4+ T cells [471]. The regulatory region of miR-34a contained NF-κB binding motifs and a pharmacological inhibitor of NF-κB, Bay 11-7082, reduced miR-34a expression, suggesting that increased expression of miR-34a in HTLV-1-infected cells may be due to Tax-mediated activation of the NF-κB pathway. Introduction of an miR-34a mimic reduces levels of the pro-apoptotic Bcl-2 family member Bax, whereas sequestration of miR-34a with a sponge construct increases cell death. These findings suggest that the increased expression of miR-34a may support the survival of HTLV-1-infected cells in part by suppressing Bax expression. MiR-146a and miR-155 are also reported to be upregulated in HTLV-1-infected cells through the Tax-mediated activation of the NF-κB pathway [472,473], and knockdown of miR-146a or miR-155 reduced growth of HTLV-1-infected cells. In liver cancer stem cells, increased expression of miR-155 targets tumor protein p53-induced nuclear protein 1 (p53INP1), thereby contributing to the growth of the cells [474]. The expression of miR-93 and miR-130b is also increased in HTLV-1-transformed T-cell lines and ATL cells [475]. Both of these miRNAs target tumor protein 53-induced nuclear protein 1 (TP53INP1), and its expression is reduced in HTLV-1-transformed cells. Accordingly, the knockdown of miR-93 and miR-130b increases TP53INP1 expression and apoptosis, implicating the involvement of miR-93 and miR-130b in proliferation and survival of HTLV-1-infected cells.

Tax also affects miRNA biogenesis by interacting with Drosha in the nucleus, facilitating its proteasome-mediated degradation [476]. Reduced expression of Drosha somehow facilitates the replication of HTLV-1.

The miRNAs among those mentioned above, which have been reported to be induced by Tax, are summarized in Table 6.

### 4.8. Epigenetic Genes

Tax also has epigenetic effects and ATL cells show characteristic epigenetic changes [477,478]. ATL genomes are characterized by the prominent methylation of CpG islands in promoter regions, and the levels of hyper methylation correlated with ATL development and progression [479]. HTLV-1-infected cells show a differentially methylated position such as *thymocyte-expressed molecule* (*THEMIS*), *leukocyte-associated immunoglobulin-like receptor 1* (*LAIR1*), and *ring-type E3 ubiquitin transferase 130* (*RNF130*), which negatively regulate T-cell receptor (TCR) signaling [479]. *Cyclin-dependent kinase inhibitor 2A* (*CDKN2A*), *Kruppel-like factor 4* (*KLF4*), *early growth response 3* (*EGR3*), and *bone morphogenetic protein 6* (*BMP6*) are also hyper-methylated in ATL cells [480,481,482]. In addition to CBP/p300, P/CAF, and HDACs, Tax interacts with multiple histone-modifying enzymes such as histone deacetylase NAD-dependent deacetylase sirtuin-1 (SIRT1), histone methyltransferases SUV39H1, and SET and MYND domain-containing protein (SMYD3) [483,484,485]. SIRT1 represses HTLV-1 viral gene expression but does not affect NF-κB-mediated transcriptional activation [483]. Binding of SUV39H1 to Tax represses the transcriptional activity of Tax, suggesting negative feedback [484]. The binding of SMYD3 to Tax enhances the Tax-mediated activation of NF-κB [485]. HTLV-1 gene expression is also under the control of epigenetic regulation. The HTLV-1 provirus genome has a CCCTC-binding factor (CTCF) binding site (CTCF-BS) at a sharp border in the *pX* region in T cells naturally infected with HTLV-1 [486]. CTCF is a zinc finger protein that binds to an insulator region in genomic DNA and plays a fundamental role in controlling higher order chromatin structure and gene expression. The binding of CTCF to the CTCF-BS of HTLV-1 provirus genome induces the hyper-methylation of DNA with repressive epigenetic marks upstream of CTCF-BS and the hypo-methylation of DNA with activating epigenetic marks downstream of CTCF-BS [486,487]. This leads to the suppression of expression of the positive strand, including Tax, while allowing the expression of the negative strand coding for HBZ. Introduction of the viral CTCF-BS into the host cellular genome also modulates cellular gene expression by forming loops between the viral CTCF-BS and cellular CTCF-BS [488,489], which may modulate host cell gene expression and facilitate transformation induced by HTLV-1 infection. Tax is able to activate transcription from the LTR, even when it is heavily methylated. The methyl-CpG-binding domain 2 (MBD2) protein interacts with Tax, and MBD2 enhances the transcriptional activity of Tax against the methylated LTR [490].

Tax-mediated induction of epigenetic regulators provides an additional means by which Tax can modulate gene transcription in HTLV-1-infected cells.

Protein arginine methyltransferase 5 (PRMT5) catalyzes the formation of monomethylarginine or symmetric dimethylarginine in a variety of proteins including histones. Symmetric methylation of histone H4 on Arg3 by PRMT5 generates repressive marks on chromatin, which recruits DNA methyltransferase DNMT3A to the promoter, followed by DNA methylation and gene silencing [491]. PRMT5 expression is upregulated during HTLV-1-mediated T-cell transformation. Knockdown of PRMT5 expression or pharmacological inhibition of PRMT5 activity in HTLV-1-infected T cells resulted in increased viral gene expression and decreased cellular proliferation [492,493], suggesting involvement of PRMT5 in HTLV-1-mediated T-cell transformation.

The enhancer of zeste homolog 2 (EZH2) is the catalytic component of Polycomb repressive complex 2 (PRC2), which methylates Lys27 of HisH3 and generates a repressive mark on chromatin. Expression of EZH2 is enhanced in HTLV-1-infected cells, likely due to the Tax-mediated activation of the EZH2 promoter through the NF-κB pathway [494,495]. PRC2-mediated trimethylation at histone H3Lys27 (H3K27me3) is enhanced in ATL cells, concomitant with ATL-specific gene expression changes that included several tumor suppressors, transcription factors, epigenetic modifiers, miRNAs, and developmental genes [495]. EZH2 enhances TGF-β signaling by facilitating the association between Smad3 and Smad4. In particular, EZH2 enhances the TGF-β-induced expression of Foxp3, the master regulator of regulatory T cells (Tregs), suggesting the involvement of EZH2 in the differentiation of regulatory T cells [495]. EZH2 is also involved in the repression of N-Myc downstream-regulated gene (NDRG) in HTLV-1-infected T cells [496].

Coactivator-associated arginine methyltransferase 1 (CARM1/PRMT4) methylates Arg17 of histone H3, which serves as an epigenetic mark for transcriptional activation. Tax associates with CARM1, and CARM1 facilitates the Tax-mediated activation of HTLV-1 LTR [497], suggesting that CARM1 enhances Tax trans-activation of the HTLV-1 LTR through a direct interaction between CARM1 and Tax. Intriguingly, Tax also activated the *CARM1* gene through the NF-κB pathway and enhances methylation of Arg17 of the histone H3 on the target gene *IL-2Rα*. Moreover, knockdown of CARM1 reduced the Tax-mediated expression of *IL-2Rα* and *cyclin D2* genes and cell cycle progression [498], suggesting that the Tax-mediated induction of *CARM1* gene expression contributes to Tax-mediated target gene expression and cell cycle promotion.

The genes among those mentioned above, which have been reported to be induced by Tax, are summarized in Table 7.

### 4.9. Genes Involved in Cellular Metabolism

Cancer cells require increased metabolism to support rapid cell proliferation.

Disialoganglioside (GD2) is expressed in tumors of neuroectodermal origin. ATL cells express GD2 after in vitro culture in the presence of IL-2, concomitant with the expression of Tax. PBLs infected with Tax-expressing retrovirus also express GD2, indicating that Tax is responsible for GD2 induction. Tax increases *β-1,4-N-acetylgalactosaminyltransferase* (*GM2/GD2 synthase*) mRNA, suggesting that the induction of GD2 by Tax is due to the new synthesis of GD2 as a result of increased transcription of the *D2 synthase* gene [494,499].

Thioredoxin (TRX) plays an important role in cellular protection against oxidative stress through thiol-reducing activity [500]. TRX was originally identified as a growth factor derived from ATL cells [501,502], which facilitates autocrine cell growth [503]. Tax-mediated apoptosis in activated T cells requires an enhanced intracellular prooxidant state [504]. Tax induces the expression of TRX by activating the TRX promoter [505]. The Tax-induced expression of TRX may contribute to reduced oxidative stress induced by Tax.

Dihydroorotate dehydrogenase (DHODH) catalyzes the fourth reaction in the de novo pyrimidine biosynthesis pathway. HTLV-1-infected T-cell lines show a higher expression of DHODH, which is enhanced by Tax. Knockdown or pharmacological inhibition of DHODH decreases HTLV-1-infected T-cell growth and apoptosis, suggesting DHODH as a potential therapeutic target for treating ATL [506].

The genes among those mentioned above, which have been reported to be induced by Tax, are summarized in Table 8.

### 4.10. Genes Involved in Viral Transmission

Cell-free transmission of HTLV-1 is inefficient and HTLV-1 is usually transmitted from cell to cell. Tax-expressing HTLV-1-infected cells show an increased expression of intercellular adhesion molecule 1 (ICAM-1, CD54) [192,507], and Tax-induction in JPX-9 cells increased the expression of ICAM-1 [191,192]. Tax activates the ICAM-1 promoter through NF-κB s and CRE-like sites [191,508,509]. ICAM-1 and its receptor LFA-1 are involved in the syncytium formation induced in the co-culture of HTLV-1-positive and HTLV-1-negative human T-cell lines [507], suggesting that induction of ICAM-1 by Tax may facilitate infection of HTLV-1 from the virus-producing cells to uninfected cells. This cell-to-cell contact, termed the virological synapse, is accompanied by the re-orientation of the microtubule-organizing center (MTOC) in the infected T cell toward the cell contact region with the noninfected target cell. Tax and Tax-induced ICAM-1 co-operate to induce re-orientation of the MTOC through the cyclic AMP-binding protein-dependent pathway and Ras-MEK-ERK signaling pathway, respectively [510].

TNF-α-induced protein 2 (TNFAIP2), also known as M-Sec, plays a critical role in the cell-to-cell transmission of the virus. When purified and briefly cultured, CD4+ T cells of HTLV-1 carriers express M-Sec, which is mediated by Tax. Knockdown or pharmacological inhibition of M-Sec reduces viral infection in multiple co-culture conditions and an in vivo mouse model of HTLV-1 infection. Knockdown or inhibition of M-Sec reduces not only plasma membrane protrusions and the migratory activity of cells, but also decreases large clusters of Gag, a viral structural protein required for the formation of viral particles. Taken together, these results suggest that M-Sec, induced by Tax, mediates efficient cell-to-cell viral infection [511].

HTLV-1-infected CD4 T cells secrete a potent chemoattractant, leukotriene B4 (LTB4), to facilitate the encounter between virus-infected and target cells. LTB4 secretion is dependent on the Tax-mediated induction of phospholipase A2γ. Inhibition of LTB4 secretion or knockdown of the LTB4 receptor on target cells reduces T-cell recruitment, cellular contact formation, and virus propagation in vitro and in vivo, indicating critical roles of LTB4 in HTLV-1 transmission [512].

HTLV-1 is also transmitted vertically via breast milk. Lactoferrin facilitates the replication of HTLV-1 in lymphocytes derived from asymptomatic HTLV-1 carriers and the transmission to cord blood lymphocytes in vitro. Lactoferrin can transactivate the HTLV-1 long terminal repeat promoter [513]. In addition, HTLV-1 infection can induce lactoferrin gene expression, and Tax trans-activates the lactoferrin gene promoter. Mutual interaction between HTLV-1 and lactoferrin would facilitate and enhance the milk-borne transmission of this virus [514].

Galectins are a class of proteins that bind specifically to β-galactoside sugars, such as N-acetyllactosamine. Galectin-1 is a galactoside-binding protein which is secreted from activated T lymphocytes and increases cell-to-cell and cell-to-matrix contact. Expression of galectin-1 is enhanced in HTLV-1-infected T cells, and Tax activates the *galectin-1* gene through the NF-κB pathway. The addition of galectin-1 increases cell fusion in a co-culture assay with HTLV-1-infected cells and potentiates the infectivity of HTLV-1 envelope pseudo-typed HIV-1 virions [515]. These observations suggest that Tax-mediated galectin-1 expression may facilitate HTLV-1 infection. Galectin-3 is a β-galactoside-binding lectin, and its expression is associated with various physiological and pathological processes, including cell growth, tumor transformation, and metastasis. Galectin-3 expression is enhanced in HTLV-1-infected human T-cell lines, and Tax activates the galectin-3 promoter through the CREB and NF-κB pathways. Galectin-3 activates IL-2 production in Jurkat T cells [516].

The viral gene product p8, a proteolytically cleaved product of p12, also plays an important role in viral transmission by increasing cellular conduits and virus transmission [12,517].

The genes among those mentioned above, which have been reported to be induced by Tax, are summarized in Table 9.

### 4.11. Genes Involved in Invasion and Infiltration

As mentioned above, since CCR4 is involved in T-cell migration into the skin, enhanced CCR4 expression in HTLV-1-infected cells may explain the frequent infiltration of ATL cells into skin [251,252].

Tax induces the expression of activated cell adhesion molecules (ALCAMs) through the canonical NF-κB pathway. ALCAM blockade or knockdown reduces the migration of HTLV-1-infected lymphocytes through a layer of endothelial cells of the blood–brain barrier (BBB), suggesting involvement of ALCAM in HAM/TSP pathogenesis [518].

Tax activates the *vimentin* gene through the NF-κB pathway [519,520]. Vimentin is an intermediate filament protein whose expression is induced upon epithelial–mesenchymal transition (EMT). The role of Tax-induced expression of vimentin in T cells is yet to be elucidated.

HTLV-1-infected T cells express the *fucosyltransferase* (*Fuc-T*) *VII* gene involved in the biosynthesis of the leukocyte sialyl Lewis X, which may be related to tissue infiltration in patients with ATL. Tax activates the Fuc-T VII promoter through the CREB pathway [521,522].

One characteristic of ATL is the infiltration of various tissues by circulating leukemic cells. Matrix metalloproteinases (MMPs), which mediate the degradation of the basement membrane and extracellular matrix, play an important role in metastasis and tumor cell dissemination. HTLV-1-infected T cells express high levels of MMP-7 and MMP-9 compared with uninfected T cells. Tax activates the *MMP-7* and *MMP-9* genes through JunD/AP-1 and NF-κB/SP-1, respectively [523,524]. Plasma levels of MMP-9 correlated with organ involvement in ATL patients, suggesting that the enhanced expression of MMP-9 may be responsible for the invasiveness of ATL cells [524]. The tissue inhibitor of matrix metalloproteinases-1 (TIMP-1) has a growth factor-like activity for erythroid cells and potentially HTLV-1-infected T cells. HTLV-1-infected cell lines express high levels of *TIMP-1* mRNA, and Tax activates the TIMP-1 promoter in a human T-cell line (Jurkat) through the AP-1 binding site [525]. Tax-mediated induction of TIMP-1 may facilitate the growth of HTLV-1-infected cells.

HTLV-1-transformed cells and ATL cells show an enhanced expression of the actin-bundling protein fascin, and Tax activates the *fascin* gene through the NF-κB pathway [526,527]. Fascin also localizes to invadopodia, membrane protrusions formed at the adherent cell surface that facilitate extracellular matrix (ECM) invasion, providing a potential molecular mechanism for how fascin increases the invasiveness of cancer cells.

Recruitment of HTLV-1-infected T lymphocytes into the central nervous system (CNS) is an essential step in the development of virus-associated neuroinflammatory diseases. Collapsin response mediator protein 2 (CRMP2), originally identified as a semaphorin-signaling transducer in the nervous system, is also present in T cells and regulates T-cell polarization and migration [528]. Infection of T-cells with HTLV-1 elevates CRMP2 expression through Tax-mediated activation and increases T lymphocyte migration [529]. These observations suggest the involvement of CRMP2 in the infiltration of HTLV-1-infected lymphocytes into various tissues.

Fibronectin (FN) is a glycoprotein, which is a major component of the extracellular matrix. HTLV-1- infected T cells show enhanced expression of FN, and Tax trans-activates the FN promoter in Jurkat cells through the NF-κB pathway [530].

The genes among those mentioned above, which have been reported to be induced by Tax, are summarized in Table 10.

## 5. Tax-Mediated Repression of Gene Expression

Tax not only activates transcription but also indirectly represses gene expression, which may have negative effects on the HTLV-1-mediated transformation.

### 5.1. Tax Inactivates the Tumor Suppressor p53

The tumor suppressors p53 and pRB play major roles in tumorigenesis, and in almost all cancers, the functions of both are disabled [531]. Tax not only inactivates pRB to facilitate cell growth but also inactivates p53 to maintain cell viability. HTLV-1-transformed cells contain higher amounts of wildtype p53, but its transcriptional activity is suppressed. Tax stabilizes and inactivates the p53 function by interfering with the activity of the N-terminal activation domain [532]. Tax-induced p53 inactivation is dependent upon the ability of Tax to induce NF-κB and can be inhibited by a constitutively active IκB mutant in the human Jurkat T-cell line. This Tax-mediated p53 inactivation is not due to p300 squelching [533,534], since the over-expression of p300 does not restore p53 activity in the presence of Tax. Tax cannot inhibit p53 function in p65/RelA knockout MEFs, indicating critical roles of p65/RelA in the Tax-mediated inactivation of p53 [535]. Intriguingly, the expression of exogenous p300 in H1299 cells allows for the full recovery of p53 trans-activation in the presence of Tax, suggesting that Tax utilizes distinct cell-type-specific mechanisms to inhibit p53 function [536]. HTLV-1-transformed cells show an interaction between p65/RelA and p53 that is increased by Tax and correlates with Tax-mediated p53 inhibition. In HTLV-1-transformed cells, p53 and p65/RelA form a complex on the p53 target murine double minute 2 (MDM2) promoter. These observations suggest that binding of the p65/RelA subunit of NF-κB to p53 compromises its transcriptional activity [537]. Tax-induced p65/RelA Ser-536 phosphorylation through IKKβ plays a crucial role in this process [538]. Tax also activates AKT and facilitates the IKKβ phosphorylation of p65/RelA, which is critical for NF-κB-mediated p53 inhibition [539]. Repression of p73β, a family member of p53, by Tax through competition for the cellular coactivator CBP/p300 is also reported [113].

### 5.2. Pro-Apoptotic Genes

Bcl-2-associated X protein (Bax) is a member of the pro-apoptotic Bcl-2 family, which facilitates apoptosis by destabilizing the mitochondrial outer membrane. Tax suppresses *Bax* gene expression through repression of the Bax promoter [540]. The *Bax* gene is a target of p53, raising the possibility that repression of the Bax promoter by Tax may be mediated, at least in part, by the suppression of p53 activity. *Bax* mRNA is a target of miR-34a, which is induced by Tax through the NF-κB pathway [471], which may also explain the reduced level of *Bax* gene expression in HTLV-1-infected cells.

HTLV-1-infected leukemic cells show reduced expressions of proapoptotic BH3-only proteins, such as the Bcl-2-interacting mediator of cell death (Bim) and BH3-interacting domain death agonist (Bid). Tax downregulates Bim and Bid expression at the transcriptional level through increased hypoxia-inducible factor-1α (HIF-1α) [541], suggesting that Tax may also promote cell survival by suppressing expression of the proapoptotic BH3-only proteins Bim and Bid.

FOXO3a is a forkhead family transcription factor which plays an important role in the induction of apoptosis by upregulating BIM and PUMA and downregulating FLIP. Expression of Tax in CD4+ T cells by the infection of HTLV-1 or lentiviral-mediated introduction induces AKT activation, leading to the phosphorylation and inactivation of FOXO3a. Inhibition of FOXO3a enables the long-term survival of a population of highly activated, terminally differentiated CD4+ T cells. Pharmacological inhibition of AKT activity compromises the persistence of terminally differentiated CD4+ T cells, suggesting the roles of Tax-mediated suppression of FOXO3a in the long-term maintenance of Tax+CD4+ T lymphocytes during the early-stage transformation of HTLV-1-infected cells [542].

N-myc downstream-regulated gene 2 (NDRG2) is a candidate tumor suppressor that is frequently downregulated in HTLV-1-infected cells. Tax induces the expression of NDRG2 through the NF-κB pathway. However, NDRG2 expression is suppressed in HTLV-1-infected cell lines and various types of ATL cells, likely due to methylation of the NDRG2 promoter. EZH2, a member of the polycomb family, is increased in ATL cells, likely through the activation of NF-κB by Tax [494,495]. EZH2 binds to the NDRG2 promoter and induces DNA methylation of the NDRG2 promoter. EZH2-mediated downregulation of NDRG2 may contribute to the enhanced activation of signaling pathways during ATL development [496].

### 5.3. Signal Transducers

HTLV-1-transformed cell lines and ATL cells show elevated levels of cAMP, a biological feature of regulatory T-cell populations [543,544]. Continued expression of Tax, but not transient expression, elevates cAMP concentrations with concomitant reduction in the cAMP-degrading phosphodiesterase 3B (PDE3B) [545]. Decreased expression of PDE3B is associated with inhibitory histone modifications at the PDE3B locus [545].

In contrast to Src family tyrosine kinases Fyn and Lyn, which are induced by Tax [339,546], Lck expression is reduced in HTLV-1-infected cells [39]. Tax decreases *Lck* mRNA levels in Jurkat T cells and inhibits Lck promoter activity through an E-box [187]. Hence, Tax transcriptionally represses *Lck* gene expression. In addition to Lck, the expression of Zap-70, which relays T-cell activation signals, is also suppressed in HTLV-1-infected T cells, and Tax suppresses Zap-70 expression in Jurkat T cells [546].

Src homology-2-containing protein-tyrosine phosphatase 1 (SHP-1), also known as protein-tyrosine phosphatase non-receptor type 6 (PTPN6), is a candidate tumor suppressor that counteracts the action of tyrosine kinases, which play central roles in growth factor signal transduction. Expression of SHP-1 is repressed in HTLV-1-transformed T-cell lines and fresh ATL cells. Consistent with this, Tax represses SHP-1 P2 promoter activity. NF-κB plays an important role in both basal P2 promoter activity and Tax-induced promoter silencing (TIPS). NF-κB dissociates from the SHP-1 P2 promoter following the binding of Tax and HDAC1, suggesting that Tax recruits HDAC1 to the SHP-1 P2 promoter and forms an inhibitory complex that results in the deacetylation and dissociation of NF-κB from the promoter and attenuation of SHP-1 expression [547]. Moreover, in HTLV-1-transformed cells, the SHP-1 P2 promoter is heavily CpG methylated and silenced [548]. These observations suggest that TIPS is mediated by dissociation of transcription factors from promoters, followed by subsequent DNA methylation.

Tax transgenic mice under the control of the HTLV-1 LTR develop neurofibroma [73]. The *neurofibromatosis type I* (*NF1*) gene is a tumor suppressor gene involved in familial cancer syndrome neurofibromatosis type I (NF1). Tax represses *NF1* gene expression through suppression of the NF1 promoter, thereby allowing the development of neurofibromas without the need for direct mutations in the *NF1* gene [549].

### 5.4. Cell Cycle Regulators

Tax directly binds to inactivates CDK inhibitors p16^Ink4a^ and p15^Ink4b^ [400,402]. In contrast, Tax inhibits p18^Ink4c^ activity by suppressing its expression. HTLV-1-infected T-cell lines express very low levels of p18^Ink4c^ compared with HTLV-1-negative cell lines [388], and Tax represses p18^Ink4c^ expression through an E-box [402]. This may facilitate the activation of CDK4 and CDK6 to promote cell cycle progression.

Tax represses the cyclin A promoter through a CREB/ATF site in ts13 adherent cells and continuously growing Jurkat T cells [550]. However, in IL-2-starved Kit 225 cells, in which Tax can promote cell cycle progression through the NF-κB pathway, Tax induces the *cyclin A* gene expression [373], consistent with the roles of cyclin A in the S-G2/M progression of the cell cycle.

### 5.5. Transcription Factors

Helicase-like transcription factor (HLTF) is a member of the SWI/SNF family, which have helicase and ATPase activities and regulate the transcription of genes by altering the chromatin structure. Tax represses HLTF transcription via EZH2 and also facilitates HLTF proteasomal degradation by direct interaction [551]. Restoring normal levels of HLTF expression enhances autophagy and increases the generation of defective virions, suggesting a role for reduced HLTF expression mediated by Tax in the normal replication of HTLV-1 [551].

Type I interferons (IFNα and IFNβ) are key effectors of the innate immune response against viral infection. Interferon regulatory factors (IRFs) play central roles in the activation of *IFN* genes. Virus infection is detected by pattern recognition receptors (PRRs), including various types of Toll-like receptors (TLRs) and activates IRFs by phosphorylation through IRF kinases, such as TANK-binding kinase 1 (TBK1), transforming growth factor beta-activated kinase 1 (TAK1) and IKKε. Apparently, contradictory observations have been reported for the effects on Tax on *IFN* gene expression. Tax activates TAK1, and knockdown of TAK1 reduces IFN-regulated gene expression [552]. Tax associates with IKKε and TBK1, which phosphorylate and activate IRF3 and enhance IFNβ promoter activity [553]. In contrast, Tax binds to and inhibits TBK1, thereby suppressing IRF3 phosphorylation and induction of the *IFN* genes [554], supporting the roles of Tax in suppression of the IFN response in HTLV-1-infected cells. ZNF268 is a zinc finger protein of the Kruppel-related family, which interacts with TBK1 to facilitate IRF3 activation during viral infection. *ZNF268* mRNA was repressed in HTLV-1-infected cells, and Tax represses ZNF268 expression through the CREB/ATF pathway [555]. Expression of Tax in Jurkat T cells induces ICSAT/Pip/LSIRF, which negatively regulates the activity of interferon-regulated genes [556], supporting the concept that Tax negatively regulates the IFN response.

B-cell CLL/Lymphoma 11B (BCL11B) is a zinc finger transcription factor which is expressed in all T-cell compartments and is indispensable for T-lineage development. HTLV-1-infected T-cell lines show a reduced expression of BCL11B, and Tax suppresses the expression of BCL11B [557], suggesting an inhibitory role for BCL11B in the oncogenesis of HTLV-1-infected T cells.

GATA-binding protein 3 (GATA3) is a transcription factor whose expression is high in naïve and Th2 T cells but low in Th1 T cells. *GATA3* mRNA expression is decreased in HTLV-1 carriers and in patients with HTLV-1-related diseases [558]. Tax suppresses the GATA3 promoter through interactions with the repressor ZEB and Sp1 [559]. Intriguingly, genetic mutations in GATA3 are reported [59,60,61], suggesting that the loss of function of GATA3 may favor the pathogenesis of ATL.

Tax represses the transcriptional activity of MyoD through competitive binding to the KIX domain of p300 with MyoD [560], providing a potential mechanism for the Tax-mediated repression of the basic helix-loop-helix transcription factors.

The c-Myb proto-oncogene is preferentially expressed in hematopoietic lineages and highly expressed in several leukemia types. The *B-myb* gene was identified on the basis of its homology with c-Myb. B-Myb plays an important role in the transition from the G1 to S phase of the cell cycle. Both Tax and c-Myb interact with the KIX domain of CBP, and Tax and c-Myb antagonize each other in transcriptional activation, suggesting that Tax suppresses the transcriptional activity of c-Myb by competing for CBP [561]. However, Tax mutant M22, which can bind to CBP, cannot repress c-Myb, and Tax mutant V89A, which cannot bind to CBP, can still suppress c-Myb, indicating that competition for CBP/p300 is not the underlying mechanism of Tax-mediated repression of c-Myb [562]. Tax suppression of c-Myb leads to the trans-repression of the c-Myb promoter through the Myb responsive elements in Jurkat T cells [562]. Not only c-Myb but also B-Myb gene expression is suppressed in HTLV-1-transformed cells, and the induction of Tax in JPX-9 results in the suppression of both the c-Myb and B-Myb gene expression [562,563]. The ability of Tax to repress c-Myb parallels that to activate NF-κB, as shown by Tax mutants. In addition, IκBα and NEMO knockout compromise the Tax-mediated repression of c-Myb. NF-κB-mediated repression of c-Myb seems to be due to the sequestration of CBP/p300 by RelA, since mutant p300, lacking the C/H1 and KIX domains, and unable to bind RelA, retains the ability to stimulate c-Myb transcription and prevents NF-κB-mediated repression [564].

### 5.6. Others

HTLV-1-infected cells show defects in DNA repair, especially base-excision repair of oxidative damage [436]. DNA polymerase β (Pol β) is involved in the base-excision repair (BER) [565], and Tax downregulated its expression [566]. A consensus bHLH binding site in the DNA polymerase β promoter mediates repression by Tax [567]. This may contribute to the increased mutation rate in Tax-expressing cells.

The *pre-T-cell receptor α* (*pTCRα*) gene encodes a polypeptide which associates with the TCRα chain and CD3 molecules to form the pre-TCR complex. The surface expression of the pre-TCR is pTCRα dependent, and signaling through this complex triggers an early αβ T-cell developmental checkpoint inside the thymus, known as β-selection. Tax suppresses the E47-mediated activation of the pTCRα promoter and the *pTCRα* gene expression, which is dependent on its ability to interact with p300. The expression of Tax in human immature thymocytes decreases the *pTCRα* gene transcription. These observations indicate that Tax, by silencing E47, downregulates *pTCRα* gene transcription during early thymocyte development [568].

The expressions of miR-149, miR-873, and miR-223 have been reported to be downregulated in HTLV-1-infected cells [472,569]. miR-149 and miR-873 target p300 and P/CAF, which play crucial roles in the Tax-mediated activation of target genes.

The genes among those mentioned above, which have been reported to be downregulated by Tax, are summarized in Table 11.

## 6. Conclusions

**Figure 6:**
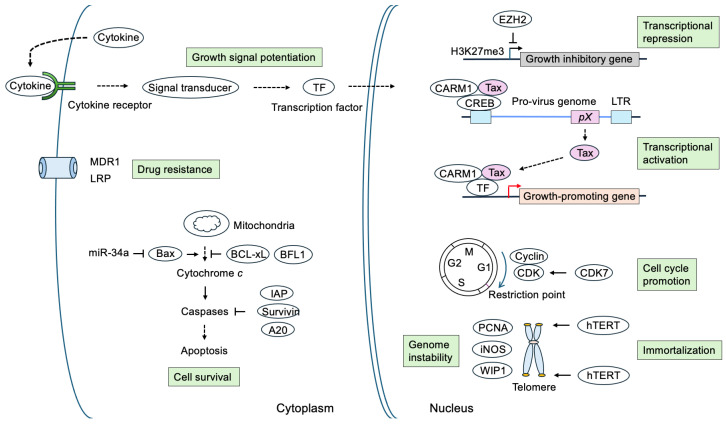
Representatives of host cell target genes regulated by Tax. The expected roles of the gene products involved in early transformation of HTLV-1-infected cells are depicted. Solid arrows indicate activation, dashed arrows indicate pathways, and ⊥ indicates inhibition.

Tax activates cellular transcription factors through the interaction with a plethora of cellular factors and induces the expression of a variety of genes involved in cell proliferation, cell survival, immortalization, immune response, invasion, genome instability, and others, thereby contributing to the early-stage transformation of HTLV-1-infected cells (Figure 6). Induction of many of the genes crucial for transformation is mediated through the NF-κB pathway. Tax also promotes transformation through protein–protein interactions. Genome instability induced by Tax leads to the accumulation of mutations, which contribute to malignant transformation.

## 7. Future Perspectives

Tax is a pleiotropic protein with multiple functions such as transcriptional activation, modulation of signal transduction pathway, promotion of cell proliferation, suppression of apoptosis and promotion of cell survival, production of ROS, induction of genomic instability, which leads to the accumulation of genetic mutations, and the induction of epigenetic changes and others. These effects co-operatively or additively contribute to the early-stage transformation of virus-infected cells by facilitating cell proliferation, survival, and immune evasion, thereby enabling persistency and the gradual increase in the virus-infected cells. These effects of Tax are exerted through the direct interaction of Tax with cellular factors and indirectly through the induction of target gene expression. Further understanding of each of these functions of Tax and identification of whole repertoire of transcriptional target genes of Tax will deepen our understanding of the mechanisms of leukemogenesis induced by HTLV-1 infection and will pave the way to develop new therapeutic approaches.

The receptors for HTLV-1 identified to date include the glucose transporter GLUT-1 [570], neuropilin 1 (NRP1) [571,572], and heparan sulfate proteoglycans [572,573,574], which are widely expressed among cell types. Consistent with this, HTLV-1 can infect cell types other than T cells, such as B cells, monocytes, endothelial cells, and fibroblasts in vitro. However, HTLV-1 infection specifically causes T-cell malignancy in vivo. The mechanism underlying T-cell-specific transformation by HTLV-1 has yet to be elucidated and would be an important issue to address in future studies. Intriguingly, there seems to be T-cell-specific effects of Tax in transcriptional regulation. Tax can promote cell cycle progression in resting T cells concomitant with the induction of cyclin D2 and CDK6 expression through the NF-κB pathway [71,373,378]. In contrast, Tax cannot induce cyclin D2 or CDK6 nor induce cell proliferation in fibroblasts, whereas the over-expression of cyclin D2 and CDK6 can induce cell cycle progression [378]. In addition, Tax inactivates p53 through the activation of NF-κB in T-cells, whereas in adherent cells, Tax acts via sequestration of CBP/p300 [535,536,537]. These observations suggest the presence of cell-type-specific effects of Tax in regulation of the NF-κB pathway. Elucidation of the entire picture of the cell-type-specific effects of Tax and the underlying mechanisms would be critical to understand cell-type-specific transformation induced by HTLV-1 infection.

## Figures and Tables

**Table 1:** Cytokines, their receptors, and cell surface molecules induced by Tax.

Gene	Gene Description	References
	Interleukins and those receptors	
IL-1α	Interleukin-1α	[254,255,256,257,258]
IL-1β	Interleukin-1β	[256,259]
IL-2	Interleukin-2	[208,209,210,211,212,213]
IL-2Rα	Interleukin-2 receptor α (CD25, Tac antigen)	[208,209,214,215,216,217,218,219]
IL-2Rγ	Interleukin-2 receptor γ (CD132, common γ chain)	[222]
IL-4	Interleukin-4	[260]
IL-5	Interleukin-5	[203,227,261]
IL-6	Interleukin-6	[188,254,256,262,263,264,266]
IL-6R	Interleukin-6 receptor	[265]
IL-8	Interleukin-8	[205,267]
IL-9	Interleukin-9	[227,266]
IL-10	Interleukin-10	[83,268]
IL-12	Interleukin-12	[270]
IL-13	Interleukin-13	[227,271,272,273]
IL-15	Interleukin-15	[229]
IL-15R	Interleukin-15 receptor	[231]
IL-17	Interleukin-17	[274]
IL17R	Interleukin-17 receptor	[275]
IL-21	Interleukin-21	[232]
IL-21R	Interleukin-21 receptor	[232]
	Chemokines and those receptors	
CCL1	C-C motif chemokine ligand 1	[286]
CCL2 (MCP-1)	C-C motif chemokine ligand 2 (monocyte chemoattractant protein-1)	[270,284,285]
CCL3 (MIP-1α)	C-C motif chemokine ligand 3 (macrophage inflammatory protein 1α)	[184,284,285,287]
CCL4 (MIP-1β)	C-C motif chemokine ligand 4 (macrophage inflammatory protein 1β)	[288,289]
CCL5 (RANTES)	C-C motif chemokine ligand 5 (regulated on activation, normal T cell expressed and secreted)	[284,290]
CCL7 (MCP-3)	C-C motif chemokine ligand 7 (monocyte chemoattractant protein-3)	[270]
CCR9	C-C chemokine receptor type 9	[198]
CCL11	C-C motif chemokine ligand 11 (Eotaxin)	[270]
CCL20 (MIP-3α)	C-C motif chemokine ligand 20 (macrophage inflammatory protein 3α)	[291]
CCL22	C-C motif chemokine ligand 22	[292]
CXCR4	C-X-C chemokine receptor type 4	[296]
CXCR7	C-X-C chemokine receptor type 7	[186]
CXCL10 (IP-10)	C-X-C motif chemokine ligand 10 (interferon γ-induced protein 10)	[184,284,293]
CXCL12 (SDF-1) (PBSF)	C-X-C motif chemokine ligand 12 (stromal cell-derived factor 1) (pre-B cell growth-stimulating factor)	[295]
XCL1	Chemokine, C motif, and ligand 1	[184]
	Growth factors	
c-sis	Platelet-derived growth factor subunit B	[226]
TGF-β1	Transforming growth factor β1	[234,235]
VEGF	Vascular endothelial growth factor	[243]
GM-CSF (CSF2)	Granulocyte–macrophage colony-stimulating factor (colony-stimulating factor 2)	[209,218,282,283]
G-CSF	Granulocyte colony-stimulating factor	[283]
NGF	Nerve growth factor	[311]
	Costimulatory molecules	
gp34	Tumor necrosis factor ligand superfamily member 4 (CD252)	[181,239,240]
OX40	Cluster of differentiation 134	[189,237]
CD40	Cluster of differentiation 40	[241]
CD40L	CD40 ligand	[242]
	Tumor necrosis factor-related	
TNF-α	Tumor necrosis factor α	[256,270,277,278,279]
TNF-β	Tumor necrosis factor β (Lymphotoxin)	[256,280,281]
TNFRSF9/4–1BB/CD137/ILA	Tumor necrosis factor receptor superfamily member 9	[307]
CD70	Tumor necrosis factor ligand superfamily member 7	[308]
	Cell adhesion molecules	
VCAM-1	Vascular cell adhesion molecule 1 (CD106)	[204,298]
E-cadherin		[306]
	Others	
PTHrP	Parathyroid hormone-related peptide	[245,246,247,248]
IFN-γ	Interferon γ	[270,271]
OPN	Osteopontin	[302]
CD44	Cluster of differentiation 44	[303]
LFA-3 (CD58)	Lymphocyte function-associated antigen, type 3 (cluster of differentiation 58)	[191]
CD69	Cluster of differentiation 69	[309]
CD83	Cluster of differentiation 83	[193]
CD137	Cluster of differentiation 137	[305]
MHC class I	Major histocompatibility complex, class I	[310]
proenkephalin		[312,313,314]
RGMa	Repulsive guidance molecule A	[297]

**Table 2:** Proto-oncogenes, transcription factors, and growth signal transducers induced by Tax.

Gene	Gene Description	References
	Proto-oncogenes	
c-Fos	v-Fos FBJ murine osteosarcoma viral oncogene homolog	[180,315,316]
c-Jun	v-jun avian sarcoma virus 17 oncogene homolog	[206,316]
c-Myc	v-Myc avian myelocytomatosis viral oncogene homolog	[320]
c-Rel	v-Rel avian reticuloendotheliosis viral oncogene homolog	[134,326]
	Transcription factors	
Fra-1	Fos-related antigen 1	[206]
JunD		[206]
Egr-1	Early growth response 1	[206,317,318]
Egr-2	Early growth response 2	[206,318]
ETR101	Immediate-early response gene 2 (IER2)	[202]
BRF1 (ZFP36L1)	Butyrate response factor 1 (Zinc finger protein 36-like 1)	[319]
p105	Nuclear factor kappa-B, subunit 1 (NFKB1)	[326]
STAT1	Signal transducer and activator of transcription 1	[332]
STAT5	Signal transducer and activator of transcription 5	[332]
IRF-4	Interferon regulatory factor 4	[333]
IRF-5	Interferon regulatory factor 5	[336]
Nur77	Nuclear receptor sub-family 4, group A, member 1 (NR4A1)	[196,201]
BCL6	B-cell/CLL lymphoma 6	[347]
	Signal transducers	
Pim-1	Serine/threonine protein kinase PIM1	[327]
Pim-3	Serine/threonine protein kinase PIM3	[327,328]
Lyn	Lyn protooncogene, Src family tyrosine kinase	[339]
JAG1	Jagged 1	[351]
Cas-L	Crk-associated substrate lymphocyte type	[185]
MAGI3	Membrane-associated guanylate kinase, WW and PDZ domains-containing 3	[358]
	Others	
EVC	Ellis Van Creveld	[343]
IκB-ζ	Inhibitor of NF-κB-ζ	[345]

**Table 3:** Cell cycle regulators induced by Tax.

Gene	Gene Description	References
CCND1	Cyclin D1	[117,376]
CCND2	Cyclin D2	[374,375,377,378]
E2F1	E2F transcription factor 1	[373,376]
CDK6	Cyclin-dependent kinase 6	[378]
CDK7	Cyclin-dependent kinase 7	[384]
ELL2	Elongation factor, RNA polymerase II, 2	[386]
CDKN1A	Cyclin-dependent kinase inhibitor 1A CDK-interacting protein 1 (CIP1) Wildtype p53-activated fragment 1 (WAF1) p21	[389,391,392]

**Table 4:** Genes related to apoptosis and cell survival induced by Tax.

Gene	Gene Description	References
Bcl-xL	Bcl2-related protein, long isoform	[407,408]
BFL1	Bcl2-related protein A1	[410]
BIRC3	Baculoviral IAP repeat-containing 3 (cIAP2, HIAP-1)	[411,412]
XIAP	X-chromosome-linked inhibitor of apoptosis	[183]
Survivin	Baculoviral IAP repeat-containing 5 (BIRC5)	[415]
c-FLIP	Cellular FLICE-inhibitory protein	[416,417,418]
A20	Tumor necrosis factor-α-induced protein 3 (TNFAIP3)	[420]
MDR1	Multidrug resistance protein 1 ATP-binding cassette sub-family B member 1 (ABCB1)Ppermeability glycoprotein (P-glycoprotein, P-gp)	[423,424,425]
LRP	Lung resistance-related protein	[426]
FasL	Fas ligand	[190]
TRAIL	TNF-related apoptosis-inducing ligand	[428]

**Table 5:** Genes involved in the suppression of DNA repair and induction of genome instability induced by Tax.

Gene	Gene Description	References
PCNA	Proliferating cell nuclear antigen	[439,440,441]
iNOS	Inducible nitric oxide synthase	[443,445]
WIP1	Wildtype p53-induced phosphatase 1 Protein phosphatase, magnesium/manganese-dependent, 1D (PPM1D)	[453]

**Table 6:** MicroRNAs (miRNAs) induced by Tax.

Gene	Target Genes	Reference
miR-146a		[472]
miR-155	Tumor protein p53-inducible nuclear protein 1 (TP53INP1)	[473]

**Table 7:** Epigenetic genes induced by Tax.

Gene	Gene Description	References
EZH2	Enhancer of zeste homolog 2	[494,495]
CARM1	Coactivator-associated arginine methyltransferase 1 Protein arginine methyltransferase 4 (PRMT4)	[498]

**Table 8:** Genes involved in cellular metabolism induced by Tax.

Gene	Gene Description	Reference
GM2/GD2 synthase	β-1,4-N-acetylgalactosaminyltransferase	[499]
TRX	Thioredoxin	[505]
DHODH	Dihydroorotate dehydrogenase	[506]

**Table 9:** Genes involved in viral transmission induced by Tax.

Gene	Gene Description	References
ICAM-1	Intercellular adhesion molecule 1 (CD54)	[191,192,508,509]
TNFAIP2	TNF-α-induced protein 2 (M-Sec)	[511]
PLA2G4C	Phospholipase A2, group IVC	[512]
Lactoferrin	Lactotransferrin (LTF)	[514]
Galectin-1	Lectin, galactoside-binding, soluble, 1 (LGALS1)	[515]
Galectin-3	Lectin, galactoside-binding, soluble, 3 (LGALS3)	[516]

**Table 10:** Genes involved in invasion and infiltration induced by Tax.

Gene	Gene Description	References
ALCAM	Activated cell adhesion molecule	[518]
Vimentin		[519,520]
FucT VII	Fucosyltransferase 7	[521,522]
MMP-7	Matrix metalloproteinase 7	[523]
MMP-9	Matrix metalloproteinase 9	[524]
TIMP-1	Tissue inhibitors of matrix metalloproteinases-1	[525]
Fascin	Actin-bundling protein	[526,527]
CRMP2	Collapsin response mediator protein 2	[529]
FN	Fibronectin	[530]

**Table 11:** Genes downregulated by Tax.

Gene	Gene Description	References
	Pro-apoptotic genes	
Bax	Bcl-2-associated X protein	[540]
Bim	Bcl-2-interacting mediator of cell death	[541,542]
Bid	BH3-interacting domain death agonist	[541]
PUMA	p53-upregulated modulator of apoptosis Bcl2-binding component 3 (BBC3)	[542]
	Signal transducers	
PDE3B	Phosphodiesterase 3B	[545]
Lck	Lymphocyte-specific protein-tyrosine kinase	[187]
Zap-70	Zeta-chain-associated protein kinase Syk-related tyrosine kinase (SRK)	[546]
SHP-1	Src homology-2-containing protein-tyrosine phosphatase 1 Tyrosine-protein phosphatase non-receptor type 6 (PTPN6)	[547]
NF1	Neurofibromatosis type I	[549]
	Cell cycle regulators	
p18^Ink4c^	Cyclin-dependent kinase inhibitor 2C (CDKN2C)	[402]
CCNA2	Cyclin A	[550]
	Transcription factors	
HLTF	Helicase-like transcription factor	[551]
ZNF268	Zinc finger protein 268	[555]
BCL11B	B-cell CLL/Lymphoma 11B	[557]
c-Myb	v-Myb avian myeloblastosis viral oncogene homolog	[562,563,564]
B-Myb	v-Myb avian myeloblastosis viral oncogene homolog-like 2	[563]
	Others	
Pol β	DNA polymerase β	[566,567]
Type I IFN	Type I Interferon	[554]
pTCRα	Pre-T-cell receptor α	[568]
	Target genes	
miR-149	p300 and P/CAF	[569]
miR-873	p300 and P/CAF	[569]

## Data Availability

No new data were created or analyzed in this study. Data sharing is not applicable to this article.

## Abbreviations

The following abbreviations are used in this manuscript:

ABCB1	ATP-binding cassette sub-family B member 1
AC	asymptomatic carrier
ALCAM	activated cell adhesion molecule
AP-1	activator protein 1
Apaf-1	apoptotic protease activating factor 1
APC^Cdc20^	Cdc20-associated anaphase promoting complex
ATF	activating transcription factor
ATL	adult T-cell leukemia/lymphoma
ATM	ataxia-telangiectasia mutated
Bax	Bcl-2-associated X protein
BBB	blood–brain barrier
BCL	B cell CLL/lymphoma
Bcl-xL	Bcl2-related protein, long isoform
BER	base excision repair
β-TrCP	β-transducin repeat-containing protein
bFGF	basic fibroblast growth factor
BFL1	BCL2-related protein A1
BH3	Bcl-2 homology 3
bHLH	basic helix-loop-helix
Bid	BH3-interacting domain death agonist
Bim	Bcl-2-interacting mediator of cell death
BIRC3	baculoviral IAP repeat-containing 3
BMP	bone morphogenetic protein
BRF1	butyrate response factor 1
BRG1	BRM/SWI2-related gene 1
bZIP	basic leucine zipper
CADM-1	cell adhesion molecule 1
CAK	CDK activating kinase
cAMP	cyclic adenosine monophosphate
CARM1	coactivator-associated arginine methyltransferase 1
Cas-L	Crk-associated substrate lymphocyte type
CBP	CREB binding protein
CCL	CC chemokine
CCR	C-C chemokine receptor
CD	cluster of differentiation
CDK	cyclin-dependent kinase
CDKN2A	cyclin-dependent kinase inhibitor 2A
c-FLIP	cellular FLICE-inhibitory protein
cMOAT	multispecific organic anion transporter, canalicular
CRE	cAMP responsive element
CREB	CRE binding factor
CRM1	chromosomal region maintenance 1
CRMP2	collapsin response mediator protein 2
CTCF	CCCTC-binding factor
CTCF-BS	CTCF binding site
CTD	C-terminal domain
CXCL	chemokine, CXC motif, ligand
CXCR	C-X-C chemokine receptor
DNA-PK	DNA-dependent protein kinase
DC	dendritic cell
DDSB	DNA double strand break
DG2	disialoganglioside
DHH	desert hedgehog
DHODH	dihydroorotate dehydrogenase
Egr	early growth response
EVC	Ellis Van Creveld
EZH2	enhancer of zeste homolog 2
FADD	fas-associated protein with death domain
FasL	fas ligand
Fbw7	F-box and WD repeat domain containing 7
FN	fibronectin
Fuc-T	fucosyltransferase
GATA3	GATA-binding protein 3
G-CSF	granulocyte colony-stimulating factor
GLUT1	glucose transporter 1
GM2/GD2 synthase	β-1,4-N-acetylgalactosaminyltransferase
GSK3β	glycogen synthase kinase-3β
HAM	HTLV-1-associated myelopathy
HBZ	HTLV-1 basic leucine zipper factor
HDAC1	histone deacetylase 1
Hh	hedgehog
HIF-1α	hypoxia-inducible factor-1α
HLTF	helicase-like transcription factor
HR	homologous recombination
hTERT	human telomerase reverse transcriptase
HTLV-1	human T-cell leukemia virus type 1
IAP	inhibitors of apoptosis
ICAM-1	intercellular adhesion molecule 1
IFN	interferon
IGSF	immunoglobulin superfamily
IHH	Indian hedgehog
IL	interleukin
IκB	inhibitor of NF-κB
IKK	IκB kinase
iNOS	inducible nitric oxide synthase
IRF	interferon regulatory factor
JAG1	jagged
JAK	Janus kinase
KID	kinase-inducible domain
KIX	KID interacting domain
KLF4	Kruppel-like factor 4
LAIR1	leukocyte-associated immunoglobulin-like receptor 1
LFA	lymphocyte function-associated antigen
LRP	lung resistance-related protein
LTR	long terminal repeat
MAGI	membrane-associated guanylate kinase, WW and PDZ domains-containing
MAP3K	mitogen-activated protein kinase kinase kinase
Mat1	menage a trois homolog 1
MBD2	methyl-CpG-binding domain 2
Mcl-1	myeloid cell leukemia sequence 1
MCP	monocyte chemoattractant protein
MDC1	mediator of DNA damage checkpoint protein 1
MDM2	murine double minute 2
MDR1	multidrug resistance protein 1
MEKK1	MAP/ERK kinase kinase 1
MHC	major histocompatibility complex, class I
MIP	macrophage inflammatory protein
miRNA	microRNA
MMP	matrix metalloproteinase
MRP1	multidrug resistance-associated protein 1
MTOC	microtubule-organizing center
NAP1	nucleosome assembly protein 1
NDRG	N-Myc downstream-regulated gene
NEMO	NF-κB essential modulator
NER	nucleotide excision repair
NF1	neurofibromatosis type I
NF-κB	nuclear factor κB
NGF	nerve growth factor
NHEJ	non-homologous end joining
NICD	notch intra-cellular domain
NIK	NF-κB-inducing kinase
NO	nitric oxide
NOD	nonobese diabetic
NOS	nitric oxide synthase
NRP1	neuropilin 1
OPN	osteopontin
ORP4L	OSBP-related protein 4
OSBP2	oxysterol-binding protein 2
p53BP1	p53-binding protein 1
PBL	peripheral blood lymphocyte
PBM	PDZ domain-binding motif
PBMC	peripheral blood mononuclear cell
P/CAF	p300/CBP-associated factor
PCNA	proliferating cell nuclear antigen
PDE3B	phosphodiesterase 3B
P-gp	permeability glycoprotein
PHA	phytohemagglutinin
PI3K	phosphatidylinositol 3-kinases
PI(3,4,5)P3	phosphatidylinositol (3,4,5)-trisphosphate
PLA2G4C	phospholipase A2, group IVC
PLCG1	phospholipase C γ 1
PMA	phorbol 12-myristate 13-acetate
PRC2	polycomb repressive complex 2
PRMT5	protein arginine methyltransferase 5
pTCRα	pre-T-cell receptor α
PTHrP	parathyroid hormone-related peptide
P-TEFb	positive transcription elongation factor
PTPN6	protein-tyrosine phosphatase, nonreceptor-type, 6
PUMA	p53-upregulated modulator of apoptosis
pX	protein coding region X
RA	rheumatoid arthritis
RANTES	regulated on activation, normal T cell expressed and secreted
RBP-Jκ	recombination signal Jκ binding protein
RGMa	repulsive guidance molecule A
RISC	RNA-induced silencing complex
RNF8	ring finger protein 8
RNF130	ring-type E3 ubiquitin transferase 130
ROS	reactive oxygen species
SCID	severe combined immunodeficiency
SHH	Sonic hedgehog
SHP-1	Src homology-2-containing protein-tyrosine phosphatase 1
SIRT1	sirtuin-1
SMYD3	SET and MYND domain containing protein
SRE	serum response elements
SRF	serum-responsive factor
STAT	signal transducer and activator of transcription
STLV	simian T-lymphotropic virus
TAB2	TAK1-binding protein 2
TAK1	TGF-β-activating kinase 1
Tax	trans-activator of pX region
TAXBP1	Tax binding protein 1
TBK1	TANK-binding kinase 1
TCF	ternary complex factor
TCR	T-cell receptor
TF	transcription factor
TFIIH	transcription factor II H
TGF-β	transforming growth factor β
THEMIS	thymocyte-expressed molecule
TIMP-1	tissue inhibitor of matrix metalloproteinases-1
TIPS	tax-induced promoter silencing
TNF	tumor necrosis factor
TNFAIP2	TNF-α-induced protein 2
TNFRSF	tumor necrosis factor receptor superfamily
TORC	transducers of regulated CREB
TPp53INP1	tumor protein p53-induced nuclear protein 1
TRAF6	TNF receptor-associated factor 6
TRAIL	tumor necrosis factor-related apoptosis-inducing ligand
Treg	regulatory T-cell
TRX	thioredoxin
TSLC1	tumor suppressor in lung cancer 1
TSP	tropical spastic paraparesis
TSS	tax-speckle structures
VCAM-1	vascular cell adhesion molecule 1
VEGF	vascular endothelial growth factor
v-onc	viral oncogene
WIP1	wildtype p53-induced phosphatase 1
XIAP	X-chromosome-linked inhibitor of apoptosis
Zap-70	ζ-chain-associated protein kinase
ZNF268	zinc finger protein 268
XPO1	exportin 1
